# Boosted Lithium-Ion Transport Kinetics in n-Type Siloxene Anodes Enabled by Selective Nucleophilic Substitution of Phosphorus

**DOI:** 10.1007/s40820-024-01428-y

**Published:** 2024-06-17

**Authors:** Se In Kim, Woong-Ju Kim, Jin Gu Kang, Dong-Wan Kim

**Affiliations:** 1 School of Civil, Environmental and Architectural Engineering, Korea University, 02841 Seoul, South Korea; 2 Nanophotonics Research Center, Korea Institute of Science and Technology, 02792 Seoul, South Korea

**Keywords:** Li-ion battery, Two-dimensional, N-type siloxene, Doping mechanism, Kinetics

## Abstract

Doped two-dimensional (2D) materials hold significant promise for advancing many technologies, such as microelectronics, optoelectronics, and energy storage. Herein, n-type 2D oxidized Si nanosheets, namely n-type siloxene (n-SX), are employed as Li-ion battery anodes. Via thermal evaporation of sodium hypophosphite at 275 °C, P atoms are effectively incorporated into siloxene (SX) without compromising its 2D layered morphology and unique Kautsky-type crystal structure. Further, selective nucleophilic substitution occurs, with only Si atoms being replaced by P atoms in the O_3_≡Si–H tetrahedra. The resulting n-SX possesses two delocalized electrons arising from the presence of two electron donor types: (i) P atoms residing in Si sites and (ii) H vacancies. The doping concentrations are varied by controlling the amount of precursors or their mean free paths. Even at 2000 mA g^−1^, the n-SX electrode with the optimized doping concentration (6.7 × 10^19^ atoms cm^−3^) delivers a capacity of 594 mAh g^−1^ with a 73% capacity retention after 500 cycles. These improvements originate from the enhanced kinetics of charge transport processes, including electronic conduction, charge transfer, and solid-state diffusion. The approach proposed herein offers an unprecedented route for engineering SX anodes to boost Li-ion storage.

**Supplementary Information:**

The online version contains supplementary material available at 10.1007/s40820-024-01428-y.

## Highlights

- Extrinsic doping is proposed as a novel route to enhance the intrinsic properties of the two-dimensional oxidized Si nanosheet (namely, siloxene) anode for Li-ion batteries.
- Fabrication of P-doped n-type siloxene is revealed to be possible through selective nucleophilic substitution of Si atoms in siloxene with P atoms.
- Due to boosted charge transport kinetics, n-type siloxene (6.7 × 10^19^ P atoms cm^-3^) exhibits the excellent storage performance (594 mAh g^-1^ after 500 cycles) even at 2000 mA g^-1^.

**Supplementary Information:**

The online version contains supplementary material available at 10.1007/s40820-024-01428-y.

## Graphical abstract

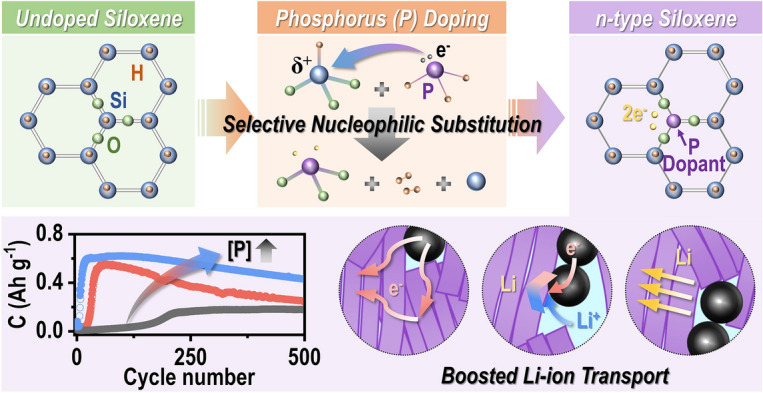

## Introduction

The escalating demand for Li-ion batteries (LIBs) with high energy and power densities has prompted the development of advanced electrode materials [1]. Si anodes hold exceptional potential in this regard owing to their huge specific capacity (~ 3580 mAh g^−1^); however, their poor cycling stabilities limits their utilization potential [2]. Two-dimensional (2D) Si-based nanosheet anodes are considered attractive in this respect because they can better accommodate volume expansion during cycling by using a layered structure with more free space [3]. These materials can be classified into three types: silicenes, siloxanes, and siloxenes [4–6]. Among them, 2D oxidized Si nanosheet, namely siloxene, has emerged as an intriguing material with wide applicability in many energy storage devices, including LIBs, Li–S batteries, and supercapacitors [3, 6–16]. There are two representative forms of siloxene depending on the crystal structure: Weiss structure (Si_6_(OH)_3_H_3_, in which alternating Si–H and Si–OH bonds are located on the surface of Si_6_ rings) and Kautsky structure (Si_6_O_3_H_6_, in which Si_6_ rings are linked through Si–O–Si bridges) [15]. The Weiss structure is metastable and progressively transforms into the Kautsky structure through the hydrolysis of Si–H and Si–OH bonds and their subsequent condensation to Si–O–Si bonds under ambient conditions [6]. A unique characteristic of Kautsky siloxenes is that a Si atom is surrounded by as many as three O atoms in the 2D plane. This renders siloxene highly hydrophilic, which, in turn, ensures good compatibility with aqueous binders for use in LIBs. This also endows siloxenes with high chemical functionality, thus expanding their material engineering potential.

The majority of previous studies on engineering Kautsky siloxene anodes for enhancing Li-ion storage have focused on incorporating secondary functional materials (i.e., controlling external factors), rather than manipulating their intrinsic properties through approaches such as doping, structure modification, or crystallinity control. Feng et al. introduced Ga–In–Sn–Zn liquid metals as self-healable matrices for siloxene during cycling [11]; however, liquid metals show a thermally unstable and chemically aggressive behavior. Qian et al. achieved a uniform solid electrolyte interphase (SEI) layer, and thus a high initial Coulombic efficiency (C.E.), by following a prelithiation approach which involves chemically lithiating siloxene prior to cycling [13]; however, the fabrication of prelithiation solutions is a complicated process and requires toxic organic solvents. Another group coated a thin layer of porous covalent organic framework (COF) onto siloxene to enhance the stability of the siloxene-electrolyte interface [12]. However, the source material (1,3,5-triformylbenzene-(tris(4-aminophenyl) amine)) used for the COF synthesis is expensive and difficult to control. A common drawback of all the aforementioned approaches is the lack of emphasis on engineering the intrinsic properties of siloxenes. Without the intrinsic control of siloxene and an understanding of how its intrinsic properties relate to Li-ion storage, it is not possible to fully harness its potential as a LIB anode and expand its applicability to other areas. Although there have been some studies on controlling the oxidation level of siloxene [7, 8, 10], which offer a feasible route for manipulating its intrinsic properties, they added SiO_x_ secondary materials for oxidation control or lacked an understanding of its intrinsic oxidation mechanism and oxidation effects.

**Fig. 1:**
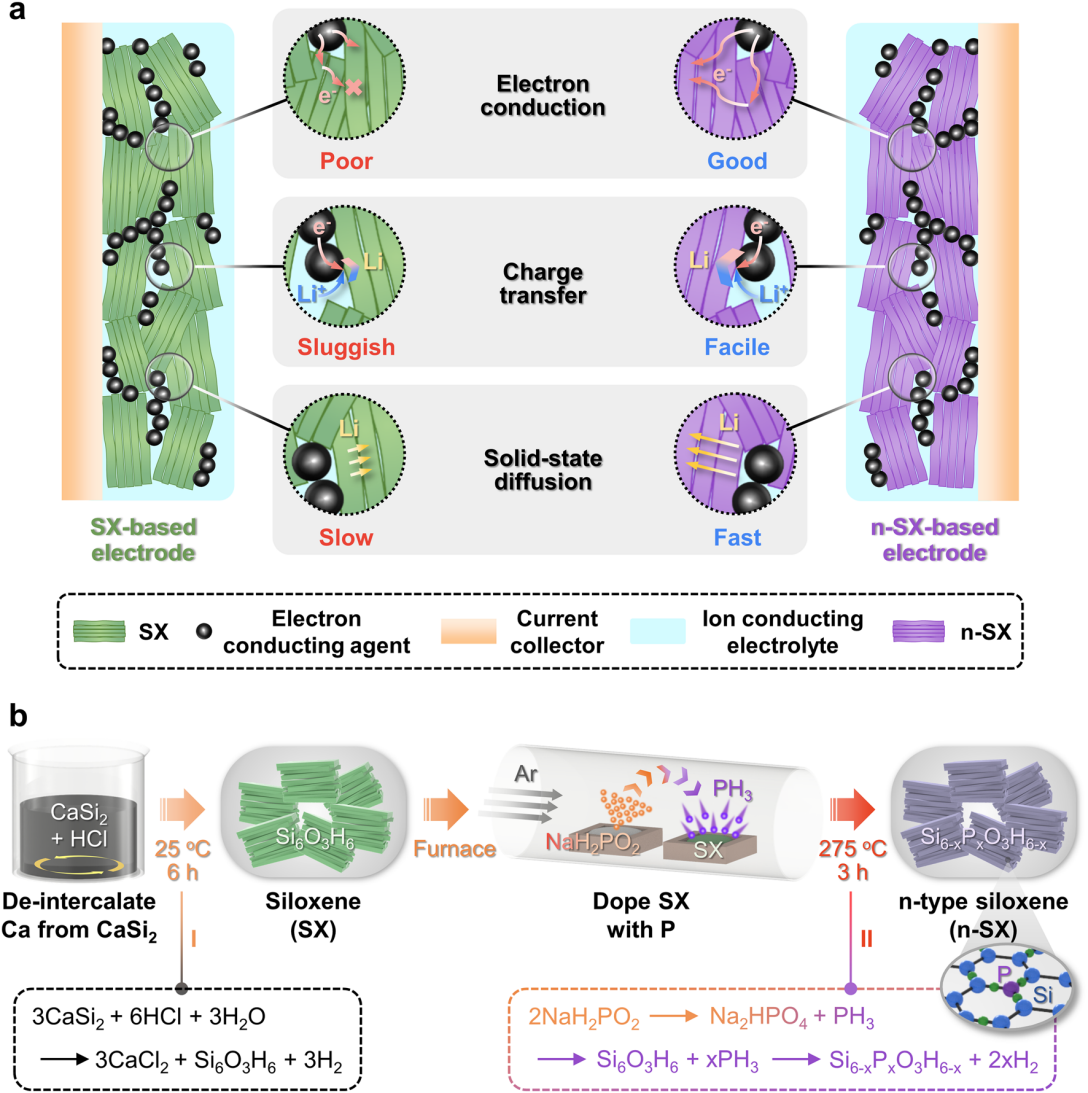
Schematics depicting the advantages of n-SX and its fabrication method. **a** Comparison of three electrochemical process rates between electrodes based on SX and n-SX. In comparison to SX, n-SX shows enhanced electron conduction, charge transfer, and solid-state diffusion capabilities. **b** Manufacturing process of n-SX begins with the de-intercalation of Ca from CaSi_2_ in an HCl aqueous solution, leading to the generation of SX. This is followed by reacting SX with PH_3_ at an elevated temperature to yield phosphorous-doped n-SX

Doping, that is, the controlled introduction of impurities into a material, is one of the most effective means of modulating its intrinsic properties [17]. This approach has long been used to manipulate the electrical, optical, and thermal properties of semiconductors used in microelectronic and optoelectronic devices [18]. In LIBs, n- or p-type Si anodes produced through doping have been proven to be effective in enhancing electrochemical performance [19–25]. However, reports of doping 2D Si-based materials, such as silicene and siloxene, with additional elements are extremely rare, presumably because of the high susceptibility of their thin, delicate, and layered morphologies to damage during the doping process. Studies exploring the effects of doping on the electronic, optical, thermoelectric, and electrochemical properties of silicene have been limited to computational approaches [26–34]. For siloxene, a recent study fabricated an N-doped siloxene/graphene composite for fiber supercapacitors; however, the doping process was performed at a very high temperature of 900 °C, resulting in the collapse of the 2D geometry and loss of the unique Kautsky crystal structure [35]. Here, an n-type siloxene (n-SX) LIB anode fabricated by doping siloxene (SX) with P atoms was successfully achieved without distorting the 2D geometry or compromising the original Kautsky-type crystal structure through a low-temperature (~ 275 °C) thermal evaporation process. This enabled the elucidation of the P-doping mechanism (i.e., selective nucleophilic substitution) using the information acquired from the measurements probing the changes in the local chemical bonds, as well as those in the Si–H vibrations of the Si_3-x_O_x_≡Si–H (0 ≤ x ≤ 3) tetrahedral building units. Based on this information, we concluded that the incorporation of P into SX leads to the generation of two, instead of one, delocalized electrons. Figure 1a illustrates the benefits of utilizing an n-SX anode over an SX anode. The increased carrier concentration in n-SX remarkably facilitated the charge transport kinetics in the three critical electrochemical processes, namely electronic conduction, charge transfer, and solid-state Li diffusion, which was experimentally validated by comparing the electrochemical performance, kinetic processes, and post-cycling chemistry of n-SX and SX. Additionally, the appropriate control of the doping concentration or the n-SX:SX ratio in the n-SX/SX mixture was revealed to enhance the electrochemical performance of the n-SX-based anodes. To the best of our knowledge, this is the first demonstration of the successful fabrication of n-SX and a systematic investigation for revealing their doping mechanism as well as the doping effect on electrochemical performance.

## Experimental Section

### Materials

CaSi_2_ (95%, technically pure), alginic acid sodium salt (alginate), and HCl (approximately 37% in H_2_O) were purchased from Sigma-Aldrich (St. Louis, MO, USA). NaH_2_PO_2_·xH_2_O was purchased from Alfa Aesar (Haverhill, MA, USA). α-polyvinylidene fluoride (PVDF) was purchased from Arkema (Colombes, France). Type-1 water (18.2 MΩ cm) was used throughout the study.

### Preparations of SX, n-SX, and Heat-Treated SX (H-SX)

#### Preparations of the SX

The SX powder was prepared via a topotactic chemical reaction. We first added 0.5 g of CaSi_2_ to 50 mL of ~ 37% HCl aqueous solution, and the mixture was stirred for 6 h at room temperature (~ 25 °C). Within a few minutes of stirring, the color of the solution changed from gray to green. After the reaction was complete, the mixture was purified by repeating eight cycles of centrifugation (8000 rpm, 5 min) and redispersion (in water). The sediments were dried in a vacuum oven at 70 °C overnight to obtain greenish powders.

#### Preparations of the n-SX

The synthesized SX and NaH_2_PO_2_·xH_2_O powders were placed into two separate alumina boats. These boats were subsequently loaded side by side into the center of the tube furnace. After purging the tube with Ar gas at a flow rate of 100 sccm for 1 h, the furnace was heated to 275 °C at a rate of 5 °C min^−1^ in an Ar atmosphere and then maintained for 3 h to allow the doping process to occur. The SX boat was positioned downstream of the Ar flow. After the reaction was terminated, the furnace was allowed to cool naturally and the n-SX powders were collected. The doping concentrations were controlled by varying the mass ratio of SX and NaH_2_PO_2_·xH_2_O from 1:2 to 1:10 and the Ar flow rate from 100 to 200 and 300 sccm. The mass of SX was maintained at 50 mg.

#### Preparations of the H-SX

The H-SX powders were prepared by following the same procedure used for the n-SX fabrication, without loading the NaH_2_PO_2_·xH_2_O powders. Briefly, the synthesized SX was loaded into an alumina boat and then placed into the center of the tube furnace. After purging the tube with Ar gas at 100 sccm for 1 h, the furnace was heated to 275 °C at a rate of 5 °C min^–1^ in an Ar atmosphere and subsequently maintained for 3 h to anneal SX. The furnace was naturally cooled down to 25 °C, and the H-SX powders were obtained.

### Material Characterizations

The sample morphologies were examined using field-emission scanning electron microscopy (SEM, SU-70, Hitachi, Tokyo, Japan), and the phases of the samples were confirmed by X-ray diffraction (XRD, Miniflex 600, Rigaku, Tokyo, Japan) using Cu Kα radiation (λ = 0.15418 nm). Raman spectroscopy (HEDA 250, WEVE, Gyeonggi, Korea) was performed at an excitation wavelength of 532 nm. The X-ray photoelectron spectroscopy (XPS) spectra were acquired using a monochromatic Al Kα source (K-Alpha^+^, Thermo Fisher Scientific, Waltham, MA, USA). The microstructure and crystallography were characterized by transmission electron microscopy (TEM, JEM-2100F, JEOL, Tokyo, Japan) at an acceleration voltage of 200 kV. Elemental mapping data were obtained using TEM equipped with an energy-dispersive X-ray spectroscopy (EDX) spectrometer. Attenuated total reflection-Fourier transform infrared (ATR-FTIR) spectroscopy (Cary 630, Agilent Technologies, Santa Clara, CA, USA) was performed in transmission mode over the range of 4000–650 cm^−1^ at a resolution of 2 cm^−1^. KBr pellets were used for IR transmittance spectroscopy. For post-cycling analysis, the samples were rinsed several times with a 1:1 v/v mixture of ethylene carbonate (EC) and diethyl carbonate (DEC) to remove any residues and then dried in an Ar-filled glove box. The Si and P contents were analyzed using ﻿inductively coupled plasma optical emission spectroscopy﻿ (ICP-OES, 5110, Agilent Technologies, Santa Clara, CA, USA).

### Electrochemical Measurements

The electrochemical properties of the electrodes were evaluated using a two-electrode coin cell assembled in an Ar-filled glovebox. Li metal (99.9%, Sigma-Aldrich) and a polypropylene membrane (Celgard 2400, Celgard, Charlotte, NC, USA) were used as the counter electrode and separator, respectively. The electrolyte was 1 M LiPF_6_ dissolved in a mixture of EC, DEC, and dimethyl carbonate at a 1:1:1 volume ratio with 10 wt% fluoroethylene carbonate additives. The working electrodes were prepared by casting a 3:1:1 (w/w/w) slurry of the active materials (i.e., SX, n-SX, or H-SX), binders, and Super P conducting agents onto a Cu foil. The electrodes were then left in vacuum at 70 °C to remove the N-methyl-2-pyrrolidone used for the PVDF binders and at 25 °C to evaporate the water for the alginate binders. The galvanostatic charge/discharge measurements and cyclic voltammetry tests were performed between 0.01 and 2.0 V vs. Li/Li^+^ using an automatic battery cycler (WBCS3000, WonATech, Seoul, Republic of Korea). Electrochemical impedance spectroscopy (EIS) measurements were conducted using an Ivium-n-Stat instrument (Ivium Technologies, Eindhoven, Netherlands) by applying an alternating current potential with an amplitude of 5 mV across a frequency range of 1 MHz to 0.01 Hz.

## Results and Discussion

### Fabrication and Material Characterizations

Figure 1b shows the fabrication schematic diagram of n-SX. First, SX was fabricated by de-intercalation of Ca^2+^ from CaSi_2_ in an HCl aqueous solution according to the following topotactic chemical reaction [6]:

1 $$3CaSi_{2} + 6HCl + 3H_{2} O \to 3CaCl_{2} + Si_{6} O_{3} H_{6} + 3H_{2}$$

Subsequently, the sodium hypophosphite hydrate (NaH_2_PO_2_·xH_2_O) and SX powders were separately placed in the furnace, and then heated at 275 °C in an Ar atmosphere. NaH_2_PO_2_ is known as an effective P doping source owing to its excellent reactivity at relatively low temperatures ranging from 250 to 300 °C [36, 37]. The reactive P precursors, that is, phosphine (PH_3_) gases, which were produced through the thermal decomposition of NaH_2_PO_2_ [38], reacted with SX to yield n-SX based on the following reactions:

2 $$2NaH_{2} PO_{2} \to Na_{2} HPO_{4} + PH_{3}$$

3 $$Si_{6} O_{3} H_{6} + xPH_{3} \to Si_{6 - x} P_{x} O_{3} H_{6 - x} + 2xH_{2}$$

The chemical formula of n-SX is Si_6-x_P_x_O_3_H_6-x_, reflecting the presence of H deficiency. We also note that P atoms are selectively substituted for Si atoms surrounded by three O atoms. These characteristics are discussed in the following sections.

**Fig. 2:**
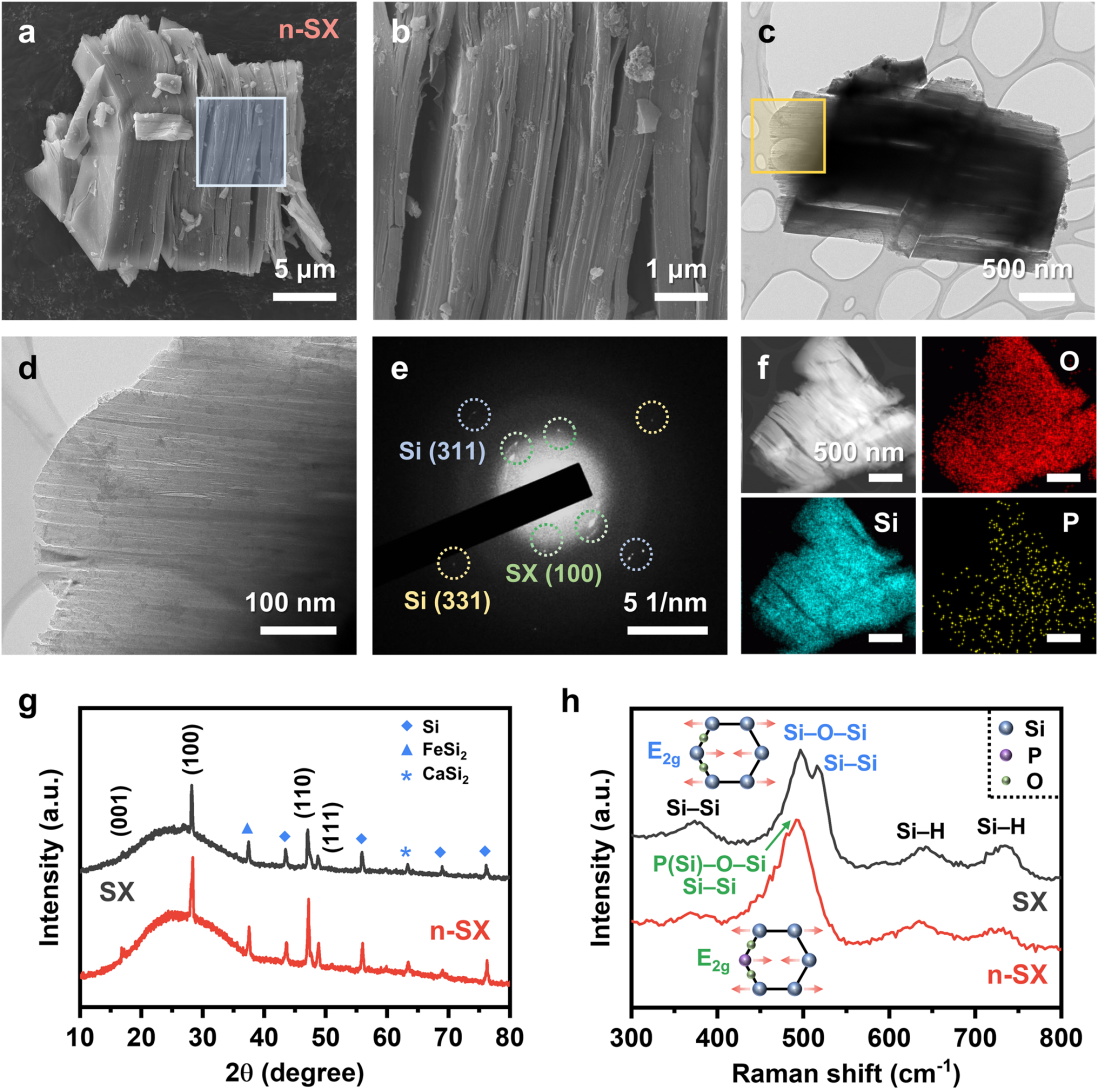
Material characterizations. **a** Low-magnification SEM image of n-SX. **b** High-magnification SEM image taken from the square in (**a**). **c** Low- and **d** high-magnification TEM images of n-SX; **d** was taken from the square in (**c**). **e** SAED pattern of n-SX with each spot indexed by their corresponding atomic planes. **f** EDX elemental mapping of O, Si, and P for n-SX. **g** 2θ/ω XRD patterns of SX and n-SX. SX peaks are indexed with their atomic planes, and impurities are with symbols. **h** Raman spectra of SX and n-SX. The E_2g_ symmetry mode is comprised of Si–O–Si and Si–Si vibrations (blue) in SX, whereas it is comprised of P–O–Si and Si–Si vibrations (green) in n-SX. Schematics of the E_2g_ modes are included

Figure 2a, b shows the SEM images of n-SX. Similar to SX (shown in Fig. S1), n-SX preserves the layered structure consisting of 2D planar sheets, indicating that the doping process carried out at the temperature used in this study (i.e., 275 °C) does not induce any morphology changes. This feature was confirmed in the low- and high-magnification TEM images of n-SX (Fig. 2c, d) and SX (Fig. S2). Both samples featured tens-of-nanometer-thick nanosheets, separated from one another by several nanometers. The thickness of a single layer of these nanosheets was presumed to fall within 0.6–1.7 nm [39, 40]. The phase and crystallography of the n-SX sample were analyzed based on its selected area electron diffraction (SAED) pattern collected over a sample area of ~ 1 μm^2^ (Fig. 2e). The strong reflections were indexed to the d-spacing (0.312 nm) of the SX (100) atomic planes, whereas the relatively weak reflections corresponded to those of Si (311) and Si (331) (0.156 and 0.127 nm, respectively). Small amounts of Si typically remain as impurities in SX produced by the de-intercalation process [40, 41]. Figure 2f displays the EDX elemental mapping of n-SX for O, Si, and P atoms. The presence of P, O, and Si was evident, demonstrating the successful incorporation of P into the SX lattice. This contradicted the EDX mapping results for SX, where P was barely detectable (Fig. S3).

Understanding the crystal structures of SX and n-SX is important to examine the effects of doping on Li-ion storage. As shown in Fig. 2g, the XRD 2θ/ω pattern of n-SX is nearly identical to that of SX. For both samples, the atomic arrangements along the [100] and [110] directions were well-ordered, whereas those along the [001] direction were poorly ordered, a frequently observed characteristic in SX [6, 10, 40, 42]. We attributed this discrepancy to the presence of sp^3^-bonded H atoms located in the out-of-plane direction, i.e., [001] [43, 44]. Both samples contain small amounts of impurities, including Si, FeSi_2_, and CaSi_2_ [40]. The local crystal structures of SX and n-SX were investigated by Raman spectroscopy (Fig. 2h). The E_2g_ vibration mode peaks differed between the two spectra: SX exhibited two peaks at ~ 496 and ~ 521 cm^−1^, corresponding to Si–O–Si and Si–Si bonds, respectively [6, 10, 45]. On the contrary, a single peak was observed at ~ 493 cm^−1^ for n-SX, stemming from the merging of the two peaks. This phenomenon was ascribed to the transformation of Si–O–Si bonds into P–O–Si bonds, which involved the breakage of Si–O bonds followed by the substitution of P for Si; this, in turn, slightly weakened the neighboring Si–Si bonds, leading to a reduction in their vibration frequencies (i.e., a smaller Raman shift). This interpretation is supported by the observation of another Si–Si peak at ~ 370 cm^−1^, with a weaker n-SX intensity than that of SX. Similarly, n-SX displayed weaker Si–H peaks at ~ 635 and ~ 730 cm^−1^ than SX, suggesting that the Si–H bonds were loosened after the doping process [46, 47].

### Identifying the Doping Sites in SX

**Fig. 3:**
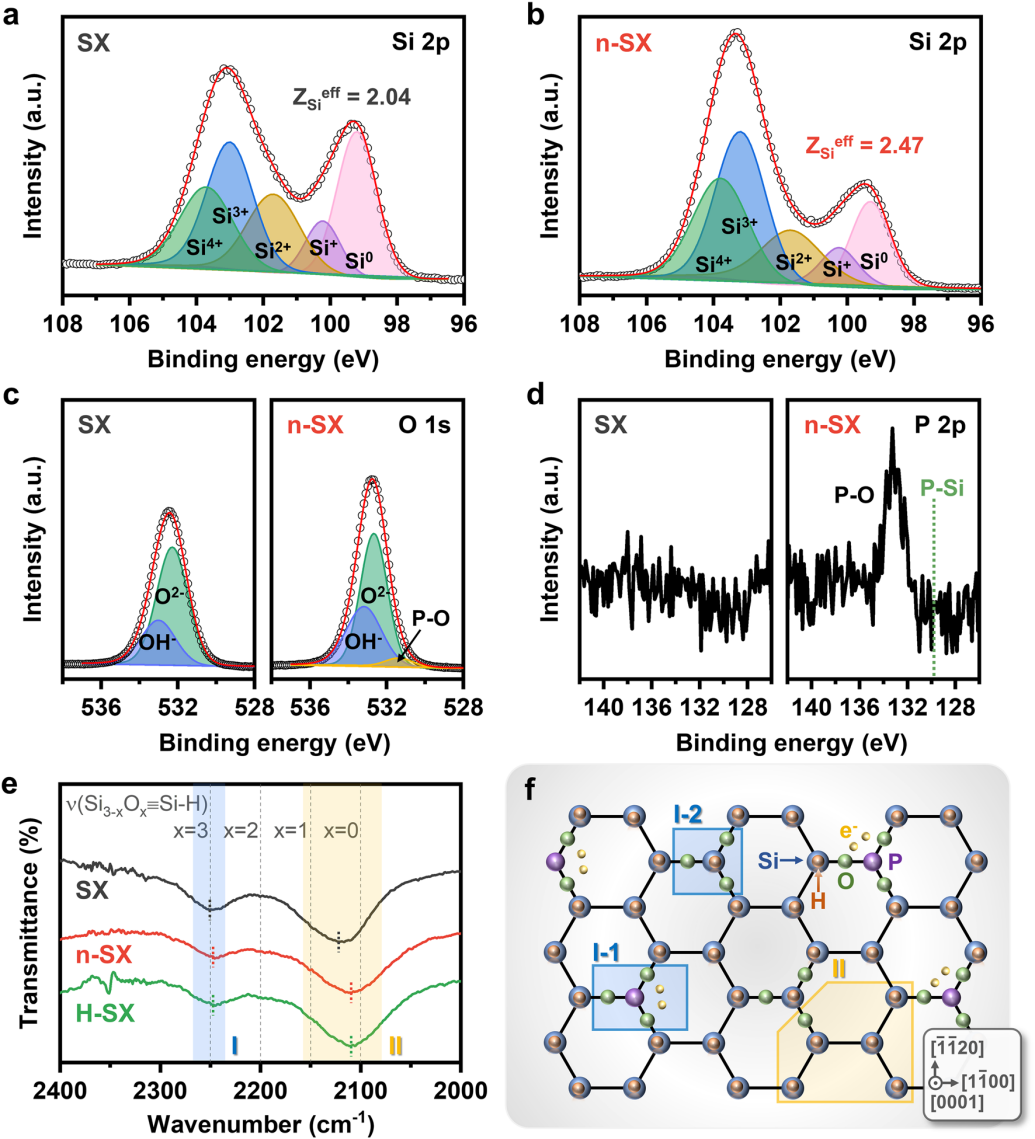
Identification of doping sites in the lattice. XPS high-resolution Si 2*p* spectra of **a** SX and **b** n-SX. Two peaks are deconvoluted into five curves associated with Si^4+^, Si^3+^, Si^2+^, Si^+^, and Si. The calculated Si effective oxidation states (Z_Si_^eff^) are denoted. XPS high-resolution **c** O 1*s* and **d** P 2*p* spectra of SX (left panels) and n-SX (right panels). The P–O bonds are confirmed in O 1*s* and P 2*p* spectra of n-SX, in contrast to SX. The P–Si bonds are not detected in P 2*p* spectrum of n-SX. **e** FTIR transmittance spectra of SX, n-SX, and H-SX. The peaks originating from the vibration modes of Si–H stretching in O_3_≡Si–H and O_x_Si_3-x_≡Si–H (0 < x < 1) tetrahedra are marked by blue (I) and orange shades (II), respectively. **f** Schematic of [0001]-projected crystal structure of n-SX. Some of O_3_≡Si–H are doped with P atoms (I-1), and the others remain undoped (I-2). None of O_x_Si_3-x_≡Si–H (0 < x < 1) are doped (II)

Although the results presented above confirm the successful incorporation of P atoms into the SX lattice, the precise locations of the doping sites remain unclear. To elucidate this, we conducted a survey (Fig. S4) using the high-resolution (Figs. 3a–d) XPS spectra of SX and n-SX. For the Si 2*p* spectra, both SX (Fig. 3a) and n-SX (Fig. 3b) showed two distinct peaks at 103.3 and 99.5 eV; however, the relative intensity between these two peaks differed significantly between the two samples. Compared to SX, n-SX exhibited a larger peak intensity (103.3 eV), whereas its intensity (99.5 eV) was smaller. To quantify this, we fitted two peaks of each sample by convoluting five sub-peaks, each centered at 103.7 (Si^4+^), 103.0 (Si^3+^), 101.7 (Si^2+^), 100.4 (Si^+^), and 99.4 eV (Si^0^) [7, 11, 12, 14]; subsequently, we estimated the effective oxidation states Z_Si_^eff^ = ΣZ_i_I_i_/ΣI_i_, where Z_i_ and I_i_ respectively denote the oxidation state and integral peak intensity of the Si^i^ species [40]. The Z_Si_^eff^ of n-SX (2.47) was ~ 1.2 times greater than that of SX (2.04), thus evidencing P doping. When P was incorporated into the SX lattice, P–Si and/or P–O–Si bonds were formed. The electronegativity (EN) of P (2.2) surpassed that of Si (1.9); thus, Si in the P–Si bonds became more positively polarized than Si in the Si–Si bonds, leading to an increased Si oxidation state. Likewise, Si in P–O–Si donated electrons to O atoms to a greater extent than Si^R^ in Si^L^–O–Si^R^ (L: left, R: right), owing to the greater capability of O in P–O–Si to attract electrons from Si, stemming from the lower EN difference (1.2) between O and P than that between O and Si (1.5); EN of O is 3.4. Figure 3c shows the high-resolution O 1*s* XPS spectra of SX and n-SX. In both spectra, a single peak was observed at 532.5 eV. For SX, this peak can be fitted by convoluting the two peaks at 533.0 and 532.3 eV, corresponding to OH^−^ and O^2−^ moieties, respectively [8, 48, 49]. In contrast, for n-SX, this peak could only be fitted when another peak at 531.4 eV for P–O bonds [50] was convoluted with the two peaks for OH^−^ and O^2−^, indicating the existence of P–O–Si bonds in the n-SX lattice. The high-resolution XPS P 2*p* spectra in Fig. 3d agreed well with the O 1*s* spectrum. No peaks were detected for SX, whereas a peak associated with the P–O bonds was observed at 133.3 eV for n-SX [51]. Notably, the n-SX spectrum did not reveal a peak for P–Si bonds at 129.9 eV [51, 52], suggesting that P substituted only Si in Si–O–Si, and not Si in Si–Si.

To further clarify the exact doping sites, we recorded the FTIR transmittance spectra of the SX, n-SX, and H-SX samples (Fig. S5); H-SX was obtained by annealing SX at the same temperature as n-SX in the absence of NaH_2_PO_2_. The largest differences among the three samples were observed in the wavenumber range of 2000–2400 cm^−1^, as depicted in Fig. 3e. The SX spectrum shows two clear valleys at ~ 2250 and ~ 2122 cm^−1^, originating from the Si–H stretching motions in the O_3_≡Si–H (region I) and Si_3-x_O_x_≡Si–H (0 < x < 1; region II) tetrahedral building units, respectively [43, 53]. These features indicated that SX formed a Kautsky-type structure. The two valleys also appeared in the H-SX spectrum, albeit with a ~ 5 cm^−1^ downward shift (~ 2245 cm^−1^) for O_3_≡Si–H and ~ 9 cm^−1^ (~ 2113 cm^−1^) for Si_3-x_O_x_≡Si–H (0 < x < 1; hereafter, abbreviated to Si_3-x_O_x_≡Si–H), compared to those in the SX spectrum. This observation indicated the weakening of the bonding strength between Si and H in both tetrahedra as a result of thermal treatment [46]. Importantly, in n-SX, the P atoms can only occupy the Si sites in O_3_≡Si–H, not those in Si_3-x_O_x_≡Si–H, indicating selective substitution. This is because only the formation of P–O–Si bonds, as opposed to P–Si bonds, was observed in the XPS spectrum of n-SX. Thus, the O_3_≡Si–H valley of the n-SX spectrum was expected to exhibit a smaller wavenumber (i.e., weaker binding strength) than that of H-SX, due to the possible formation of some O_3_≡P–H units in n-SX; this observation agreed well with the EN difference between H and P (0) being smaller than that between H and Si (0.3). However, n-SX displayed the O_3_≡Si–H valley at the same wavenumber (~ 2245 cm^−1^) as that in H-SX, contradicting our expectations. This behavior was attributed to the formation of O_3_≡P units containing H vacancies atop P (i.e., vanished P–H stretching motion), rather than the formation of O_3_≡P–H units. Unlike O_3_≡Si, which contains radicals, O_3_≡P can be stable owing to the presence of an electron pair unbound to O atoms. It can thus be concluded that, after doping, some portions of O_3_≡Si–H units that were substituted with P atoms transformed into O_3_≡P units (I-1 in Fig. 3f), while the remaining O_3_≡Si–H units (I-2 in Fig. 3f) exhibited loosened Si–H bonds. Meanwhile, the Si_3-x_O_x_≡Si–H wavenumber for n-SX (~ 2113 cm^−1^) remained invariant relative to that for H-SX, indicating that Si–H bond weakening in Si_3-x_O_x_≡Si–H occurred to the same degree in both cases, due to exposure to the same annealing temperature. The Si_3-x_O_x_≡Si–H building unit in n-SX is marked by II in Fig. 3f. Our findings suggest that Si_6-x_P_x_O_3_H_6-x_ possesses the chemical formula n-SX.

### P Substitution and Electron Generation Mechanisms

**Fig. 4:**
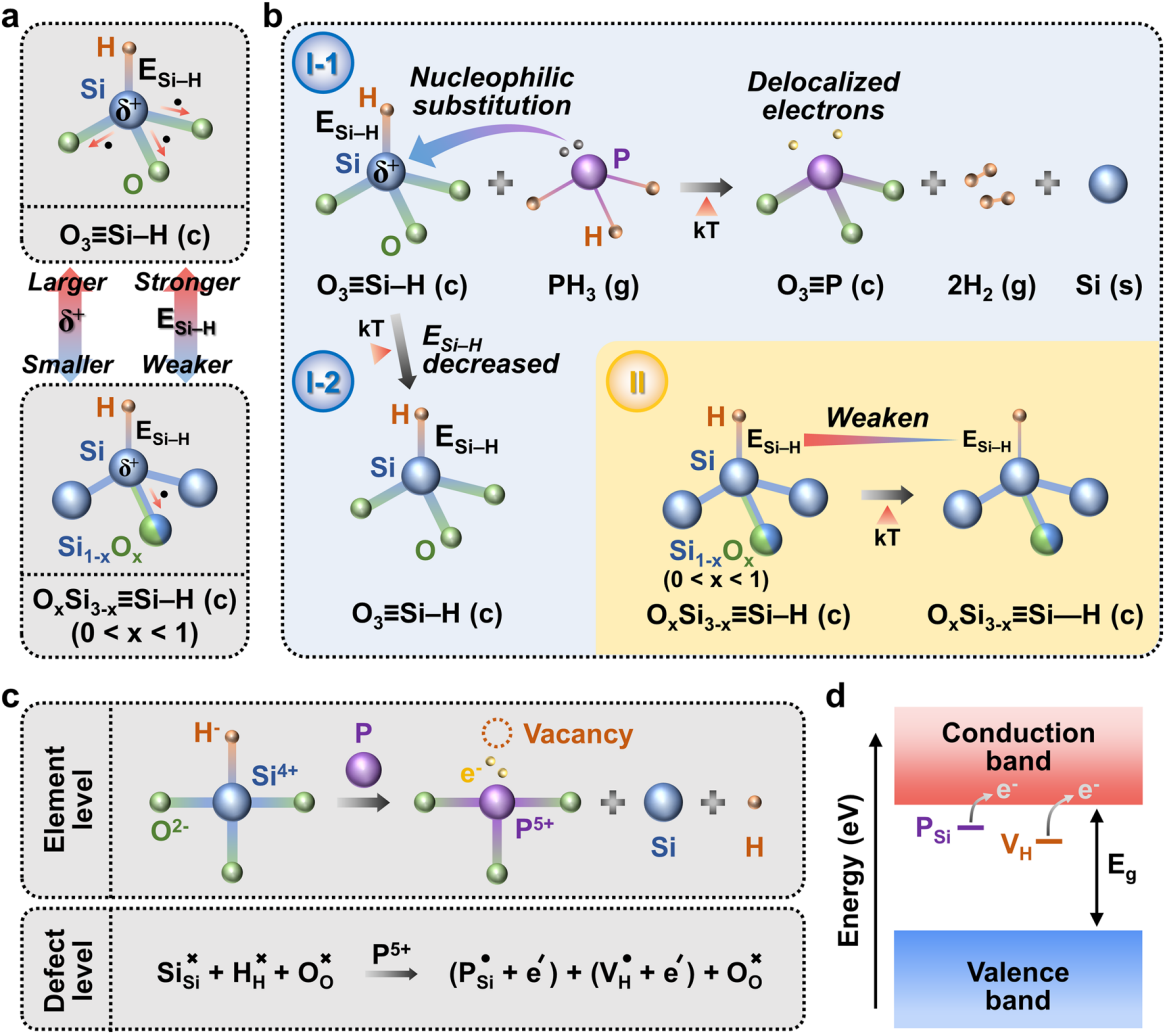
Mechanisms of P-doping and two-electron generation. **a** Comparisons of δ^+^ and E_Si-H_ between O_3_≡Si–H and O_x_Si_3-x_≡Si–H tetrahedra. The former exhibits larger δ^+^ and stronger E_Si-H_ than the latter. **b** Reactions occurring when P sources are supplied to SX in the presence of thermal input (kT). Si atoms in some O_3_≡Si–H are substituted with P atoms via nucleophilic reaction, creating PO_3_ units with two delocalized electrons (I-1). Unreacted O_3_≡Si–H remains intact due to strong E_Si-H_ (I-2). O_x_Si_3-x_≡Si–H (0 < x < 1) do not participate in doping, but their E_Si-H_ becomes weaker owing to kT (II). The acronyms, c, g, and s, denote crystal, gas, and solid, respectively. **c** Mechanism of two-electron generation achieved by P-doping. The upper diagram describes at the (charged/neutral) element level, while the lower one illustrates at the defect level using Kröger–Vink notation. **d** Energy band diagram of n-SX. The two types of defect sites form the donor levels close to the conduction band, generating two free electrons. *E*_g_ denotes the energy band gap, known to be 2.5 eV for SX

Thus far, it has been established that P selectively substitutes Si in O_3_≡Si–H units rather than Si in Si_3-x_O_x_≡Si–H units. Figure 4a shows the molecular structures of the two units. The degree of positive polarization (δ^+^) of Si in O_3_≡Si–H exceeds that of Si in Si_3-x_O_x_≡Si–H, because Si in O_3_≡Si–H is surrounded by three O atoms with a high EN value (3.4), whereas Si in Si_3-x_O_x_≡Si–H is surrounded by either zero or one O atom. This aspect results in the greater electron deficiency of Si in O_3_≡Si–H relative to that of Si in Si_3-x_O_x_≡Si–H. Thus, during doping, the nucleophilic PH_3_ precursors are likely to selectively attack Si in O_3_≡Si–H, leading to the formation of O_3_≡P as follows:

4 $$O_{3} \equiv SiH(c) + PH_{3} (g) \to O_{3} \equiv P(c) + 2H_{2} (g) + Si(s)$$

where *c*, *g*, and *s* denote the crystal unit, gas, and solid states, respectively (I-1 in Fig. 4b). Notably, one substituted P atom creates two delocalized electrons in n-SX, in contrast to the single-electron generation process that occurs in the doping process of Si, graphite, and graphene [19, 54, 55]. This anomaly was ascribed to the presence of H vacancies in n-SX. Figure 4c illustrates the two-electron generation mechanism from the perspective of defect chemistry. The element-level diagram (top) can be converted into the defect-level diagram (bottom), which represents the substitution reaction, i.e., O_3_≡Si–H + P → O_3_≡P + Si + H, using the Kröger–Vink notation [56]:

5 $$Si_{Si}^{ \times } + H_{H}^{ \times } + O_{O}^{ \times } \mathop{\longrightarrow}\limits^{{P^{5 + } }}(P_{Si}^{ \bullet } + e^{\prime}) + (V_{H}^{ \bullet } + e^{\prime}) + O_{O}^{ \times }$$

Upon substitution, the difference in the oxidation state between P (+ 5) and Si (+ 4) results in the formation of $P_{Si}^{ \cdot }$ with an effective + 1 charge, which is compensated by one free electron ($e^{\prime}$) with an effective -1 charge. In addition, an H vacancy ($V_{H}^{ \cdot }$) with an effective + 1 charge, which is created atop P, requires another electron for compensation. Thus, two free electrons were generated in n-SX. Figure 4d shows the energy-band diagram of n-SX. Inside the band gap (~ 2.5 eV) [57], two types of donors, namely $P_{Si}$ (the P atom residing on the Si site) and $V_{H}$ (the H vacancy), may be located in the vicinity of the conduction band at shallow and deep levels, respectively, with the extrinsic donors tending to be closer to the conduction band than the intrinsic donors [58, 59].

The larger δ^+^ of Si in O_3_≡Si–H than that of Si in Si_3-x_O_x_≡Si–H is mainly responsible for the Si–H binding strength (E_Si–H_) difference between these two cases. The dipole moment formed between Si and H in O_3_≡Si–H surpasses that in Si_3-x_O_x_≡Si–H, resulting in a stronger E_Si–H_ for O_3_≡Si–H compared to that for Si_3-x_O_x_≡Si–H (Fig. 4a) [60]. Therefore, when subject to a thermal energy of kT, where k is the Boltzmann constant and T is the absolute temperature in K (~ 548 K in this work), the E_Si–H_ of O_3_≡Si–H weakens to a smaller extent (I-2 in Fig. 4b) compared to that of Si_3-x_O_x_≡Si–H (II in Fig. 4b). This explains the less pronounced valley shift observed in the FTIR spectra of n-SX and H-SX (Fig. 3e) for O_3_≡Si–H (~ 5 cm^−1^), compared to that for Si_3-x_O_x_≡Si–H (~ 9 cm^−1^). It is important to point out that the Si–H bonds were preserved in n-SX, as they play crucial roles in forming stable intermolecular linkages with aqueous binders [40, 61]. These bonds sever under annealing temperatures exceeding 400 °C (~ 673 K) [46, 47].

### Li-Ion Storage Performance and Electrochemical Kinetics

**Fig. 5:**
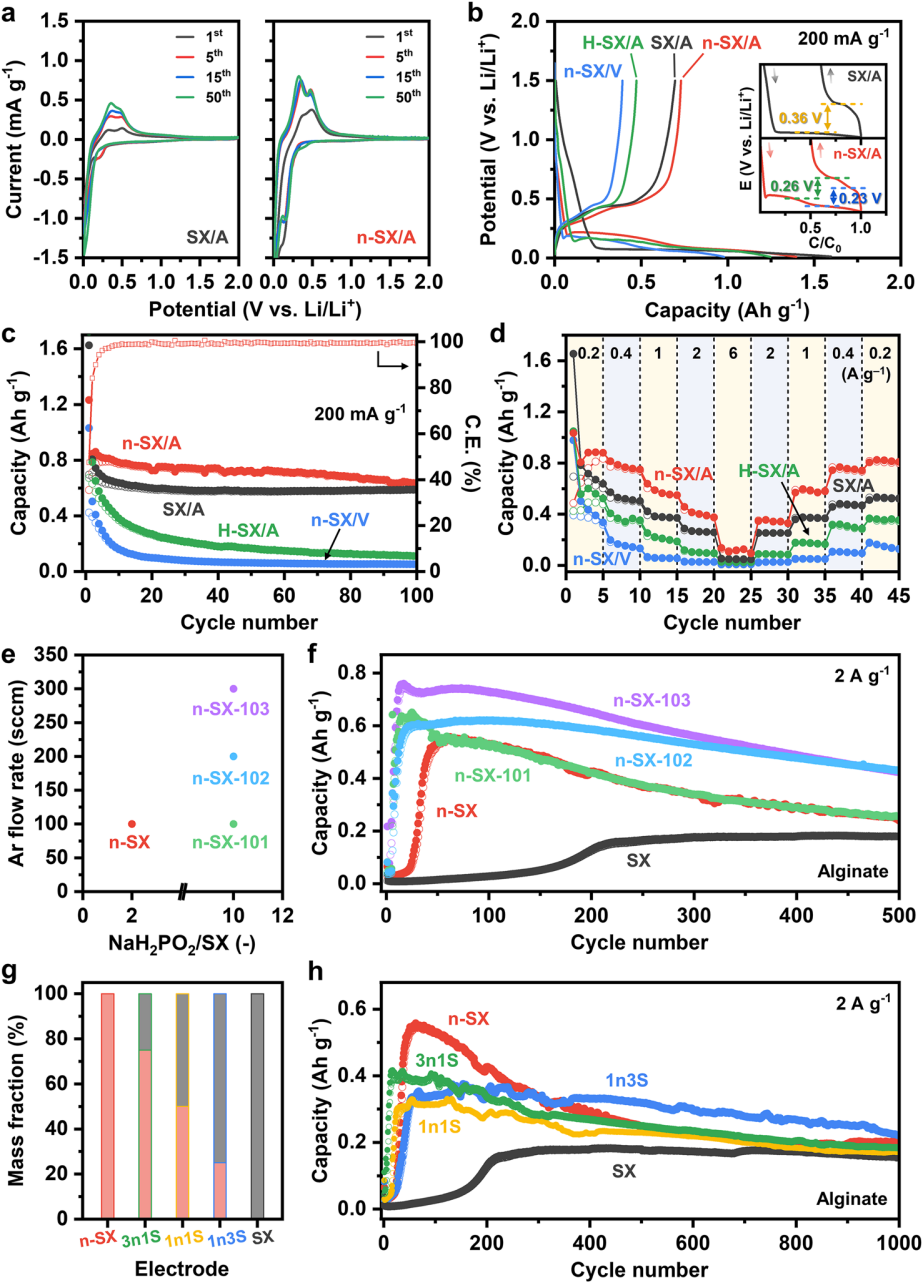
Electrochemical performance of the electrodes. **a** CV curves of SX/A (left) and n-SX/A collected at 0.1 mV s^−1^ during the first, 5th, 15th, and 50th cycles of cathodic/anodic scans. **b** Galvanostatic charge/discharge voltage curves at 200 mA g^−1^ for SX/A, n-SX/A, n-SX/V, and H-SX/A electrodes. Inset shows the voltage curves of SX/A and n-SX/A after a horizontal translation followed by a horizontal inversion were applied to their charge curves. The polarization values (ΔE) for each lithiation/delithiation pair are presented. **c** Cycling stability of the four electrodes at 200 mA g^−1^ over 100 cycles. The corresponding C.E. of n-SX/A is also plotted using the right-hand axis. **d** Rate performance evaluated by incrementally increasing the current densities from 0.2 to 0.4, 1, 2, and 6 A g^−1^, followed by reversing the order. **e** Two experimental parameters used for varying the doping concentrations of n-SX. The mass ratios of NaH_2_PO_2_ to SX and the Ar flow rates are 2:1 and 100 sccm for n-SX, 10:1 and 100 sccm for n-SX-101, 10:1 and 200 sccm for n-SX-102, and 10:1 and 300 sccm for n-SX-103. **f** Cycling stability of n-SX, n-SX-101, n-SX-102, and n-SX-103 at 2 A g^−1^ over 500 cycles. **g** Compositions of the five n-SX/SX mixture electrodes. The mass ratios between n-SX and SX are 1:0 for n-SX, 3:1 for 3n1S, 1:1 for 1n1S, 1:3 for 1n3S, and 0:1 for SX. **h** Cycling stability of the five mixture electrodes at 2 A g^−1^ over 1000 cycles. The alginate binders were used

We investigated the effects of doping on the electrochemical performance of four types of electrodes: SX mixed with alginate binders (SX/A), n-SX/A, H-SX/A, and n-SX with PVDF binders (n-SX/V). Figure 5a shows the cyclic voltammetry (CV) curves of SX/A and n-SX/A at 0.1 mV s^−1^ between 0.0 and 2.0 V vs. Li/Li^+^. During the first cathodic scan, the CV of SX/A exhibited a small peak at 0.22 V, followed by a dramatic drop from 0.18 to 0.01 V, suggesting the occurrence of two-step lithiation [11]. During the first anodic scan, delithiation also proceeded through a two-step reaction occurring at 0.32 and 0.49 V, respectively [11]. These cathodic and anodic peaks increased progressively with cycling, originating from electrode activation [62, 63]. Both n-SX/A and SX/A demonstrated comparable CV behaviors. For both the cathodic and anodic processes, two-step reactions occurred over the entire cycling: between 0.17 and 0.01 V for lithiation, and between 0.32 and 0.48 V for delithiation, agreeing well with the corresponding results for SX/A. However, the current densities at each cycle for n-SX/A notably surpassed those for SX/A, implying a higher electrochemical activity of n-SX/A relative to SX/A. This improvement can be attributed to the increased electrical conductivity achieved by P-doping [18]. The CV curve of H-SX/A supports this interpretation (Fig. S6). In the CV curves of H-SX/A, two peaks appeared during both the lithiation and delithiation stages; however, the current density decreased remarkably after five cycles, falling far below those of n-SX/A and SX/A. This behavior indicated that despite the loosening of Si–H bonds in H-SX and n-SX owing to high-temperature exposure, which degrades electrochemical activity, this degradation can be significantly overcome by increasing the carrier concentration. Among the four electrodes, the lowest CV activity was observed for n-SX/V (Fig. S6), which was attributed to the dipole–dipole repulsion between Si and H in n-SX and C–F in PVDF, as well as the global nonpolar characteristic of PVDF [40].

The galvanostatic charge/discharge (GCD) voltage curves of the four electrodes at 200 mA g^−1^ during the first cycle are shown in Fig. 5b. Despite the discharge capacity of n-SX/A (1290 mAh g^−1^) being below that of SX/A (1590 mAh g^−1^), the charge capacity of n-SX/A (730 mAh g^−1^) was higher than that of SX/A (700 mAh g^−1^). The reason for this behavior was attributed to the limited formation of an irreversible solid electrolyte interphase (SEI) layer in n-SX/A, compared to SX/A [61]. The effect of doping can be more clearly revealed by comparing the polarizations (ΔE) of n-SX/A and SX/A; ΔE is defined as the difference between the delithiation and lithiation potentials [14]. Figure S7a, b shows the differential capacity (dQ/dE) plots of SX/A and n-SX/A, respectively, as acquired from the GCD curves in Fig. 5b. For SX/A, one lithiation peak at 0.01 V and one delithiation peak at 0.44 V were observed. In contrast, for n-SX/A, two peaks appeared at 0.18 and 0.08 V during lithiation, and two additional peaks were observed at 0.31 and 0.44 V during delithiation. Using these potential values, the ΔE of the two electrodes was estimated (inset of Fig. 5b). Importantly, the ΔE values of n-SX/A were 0.26 V for the SX ↔ Li_y-ζ_SX (0 < ζ < y) transition and 0.23 V for the Li_y-ζ_SX ↔ Li_y_SX transition, whereas the ΔE of SX/A was as large as 0.36 V for the Si ↔ Li_y_SX transition. The smaller ΔE of n-SX/A compared to that of SX/A represents the occurrence of faster Li insertion/extraction processes through two-step reactions, presumably due to the enhanced electrical conduction enabled by doping. Overall, the capacity increased in the order n-SX/V, H-SX/A, SX/A, and n-SX/A, which was in good agreement with the CV results. The cycling stabilities of the four electrodes at 200 mA g^−1^ and their corresponding C.E. are presented in Figs. 5c and S8. n-SX/A retained ~ 86% (630 mAh g^−1^) of its initial charge capacity (730 mAh g^−1^) after 100 cycles, while attaining a C.E. of approximately 99%. The capacity retention of SX/A after 100 cycles was ~ 84% (i.e., 590 mAh g^−1^), nearly identical to that of n-SX/A. The origin of this similarity may be due to the weaker Si–H bonds in n-SX than in SX, which in turn induced weaker intermolecular interactions with the alginate binders [40]. These weaker intermolecular bonds in n-SX/A become compromised more easily by continuous cycles of lithiation and delithiation compared to the stronger bonds in SX/A. By the 100th cycle, the drawbacks of n-SX/A begin to outweigh its advantages, such as exceptional transport kinetics and storage capabilities. Consequently, the cycling stability of n-SX/A degrades more rapidly than that of SX/A with further cycling. In contrast to stable capacity retention of n-SX/A and SX/A, H-SX/A and n-SX/V maintain only ~ 17% and ~ 12% of their initial capacities after 100 cycles, respectively. This result further confirms the significance of strong intermolecular interactions between Si–H bonds and alginate binders, as well as high carrier concentration, in ensuring good cycling stability. Additionally, the capacity of n-SX/A consistently surpassed those of SX/A, H-SX/A, and n-SX/V during cycling, demonstrating its excellent performance. It should be mentioned here that n-SX/A achieved the C.E. of ~ 99% within ~ 10 cycles, whereas it takes ~ 40 cycles for SX/A and H-SX/A. This observation indicates that the delithiation processes in the n-SX electrodes occur more efficiently than those in the undoped SX electrodes due to the enhanced transport kinetics of Li-ions and electrons facilitated by P doping. Figure 5d exhibits the rate performance of the electrodes at 200, 400, 1000, 2000, and 6000 mA g^−1^. In accordance with the CV results and cycling stability, the rate performance increased in the following order: n-SX/V < H-SX/A < SX/A < n-SX/A. Even at 2000 mA g^−1^, n-SX/A delivered a capacity as high as 460 mAh g^−1^.

To boost the electrochemical performance of n-SX and understand the effects of doping concentration on Li-ion storage at a very high current density, we fabricated three other n-SX samples with different doping levels. Figure 5e shows the two experimental parameters that were varied to control the P contents in n-SX, namely the mass ratio of NaH_2_PO_2_·xH_2_O to SX (NaH_2_PO_2_·xH_2_O/SX) and the Ar flow rate. A higher NaH_2_PO_2_·xH_2_O/SX mass ratio can increase the amounts of PH_3_ species delivered to n-SX, while a higher Ar flow rate can reduce the mean free paths of the precursor gas molecules by promoting the collisions of these molecules with the Ar gas molecules, leading to a substitution reaction occurring at a higher probability. In this work, the NaH_2_PO_2_·xH_2_O/SX and Ar flow rates were respectively 2 and 100 sccm for n-SX, 10 and 100 sccm for n-SX-101, 10 and 200 sccm for n-SX-102, and 10 and 300 sccm for n-SX-103. Using the Si and P contents characterized by ICP-OES, we estimated the P dopant concentrations (in atoms cm^−3^) of the four samples (see Supplementary Note S1) as: 2.4 × 10^19^ atoms cm^−3^ for n-SX, 3.6 × 10^19^ atoms cm^−3^ for n-SX-101, 6.7 × 10^19^ atoms cm^−3^ for n-SX-102, and 8.1 × 10^19^ atoms cm^−3^ for n-SX-103 (Table S1). Figure 5f shows the cycling properties of the five electrodes, that is, SX, n-SX, n-SX-101, n-SX-102, and n-SX-103, at 2000 mA g^−1^ over 500 cycles; alginate binders were used for each case. The most remarkable enhancement observed in the doped SX electrodes, compared to their undoped counterparts, was the significantly accelerated activation process. Activation was completed after 230 cycles for SX, whereas it was achieved within 60 cycles for n-SX. For the electrodes with higher doping concentrations, activation occurred even more rapidly: 25 cycles for n-SX-101, 25 cycles for n-SX-102, and 15 cycles for n-SX-103. This behavior strongly supports the enhanced transport kinetics of Li ions and electrons [13], which was further confirmed by comparing the storage capacities of the electrodes. The post-activation capacity of n-SX (552 mAh g^−1^) was a factor of 3.6 larger than that of SX (153 mAh g^−1^). For electrodes with higher doping levels, this increase was more pronounced: 4.1 times for n-SX-101 (621 mAh g^−1^), 4.0 times for n-SX-102 (594 mAh g^−1^), and 4.9 times for n-SX-103 (756 mAh g^−1^). Particularly, n-SX-102 demonstrated excellent capacity retention, maintaining 73% (equivalent to 431 mAh g^−1^) of its post-activation capacity after 500 cycles. From the comparative analysis of the electrochemical properties of the five electrodes, it was evident that although higher doping concentrations improve carrier transport kinetics and storage capacity, excessive doping adversely affects cycling stability, underscoring the importance of optimization; as such, n-SX-102 was overall highlighted as the best electrode in this study.

While undoped SX ensures cycling stability, doped SX enhances the transport kinetics and storage capacity. This indicates that the two electrodes exhibited a complementary relationship, each with an enhancing aspect and a limitation. Therefore, we fabricated three mixed electrodes comprising n-SX and SX in mass ratios of 3:1 (3n1S), 1:1 (1n1S), and 1:3 (1n3S) (Fig. 5g), and then evaluated their electrochemical properties at 2000 mA g^−1^ over 1000 cycles (Fig. 5h). As expected, by increasing the n-SX fraction, the cycle number required for activation decreased, and the post-activation capacity tended to increase: 60 cycles and 329 mAh g^−1^ for 1n3S, 30 cycles and 315 mAh g^−1^ for 1n1S, and 14 cycles and 413 mAh g^−1^ for 3n1S. Additionally, a decrease in the n-SX fraction resulted in an increase in cycling stability. 3n1S retained 45% of its post-activation capacity after 1000 cycles, whereas this capacity retention improved to 56% for 1n1S and further increased to 67% for 1n3S. Notably, an unexpected trend was that 1n1S and 3n1S completed their activation processes much earlier than n-SX, which required 60 cycles. This suggests that the physical incorporation of SX into n-SX in appropriate proportions can enhance the transport kinetics of charge carriers, likely because of the promoted intermolecular interactions between the Si and H bonds and the OH groups in the alginate binders [40]. Overall, the use of mixed electrodes is effective in mitigating the capacity decay typically observed in doped SX electrodes, while maintaining their transport kinetics and storage capabilities without significant degradation. Table S2 summarizes the key properties of the eight electrodes, including the number of cycles required for activation, post-activation capacity, and capacity retention. As displayed in Table S3, n-SX-102 exhibited a capacity and retention comparable to or much higher than those of various SX electrodes that had been engineered intrinsically or externally in earlier studies.

**Fig. 6:**
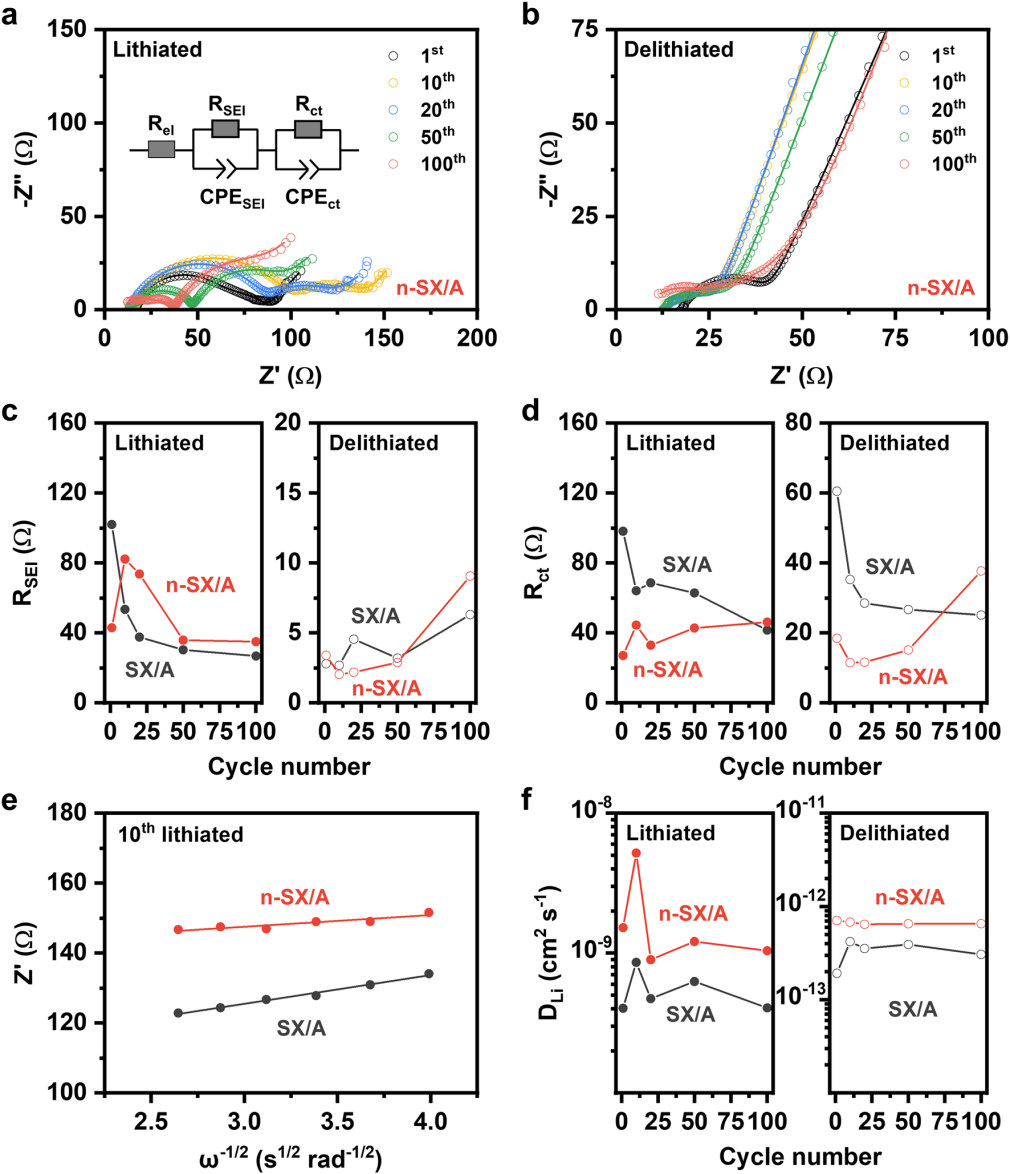
Electrochemical impedance spectroscopy. Nyquist plots of n-SX/A at its **a** lithiated and **b** delithiated states during first, 10th, 20th, 50th, and 100th cycles. Open circles and solid lines denote the measured data and fitted results, respectively. Fitted values of **c** *R*_SEI_ and **d** *R*_ct_ for SX/A and n-SX/A at their lithiated (left panels of each figure) and delithiated (right panels) states as functions of cycle number. **e** Plots of Z’ vs. ω^−1/2^ for SX/A and n-SX/A at their 10th lithiated states. Measured data (closed circles) are fitted with linear-regression (solid lines). **f** D_Li_ of SX/A and n-SX/A at their lithiated (left panel) and delithiated (right panel) states as functions of cycle number. Z’ vs. ω^−1/2^ plots are used for calculating diffusion coefficients

To elucidate the origin of the differences in the electrochemical performances of the doped and undoped electrodes, we analyzed the electrochemical kinetics of n-SX/A and SX/A using EIS analysis. Figure 6a, b displays the Nyquist plots of n-SX/A in its lithiated and delithiated states, respectively, during the first, 10th, 20th, 50th, and 100th cycles. At the lithiated state (Fig. 6a), two semicircles appeared in the high- (~ 600 Hz) and mid- (~ 2 Hz) regions, followed by a linear segment in the low-frequency region (0.5–0.01 Hz). The semicircles in the high- and mid-frequency regions were attributed to the resistances of the SEI layers (*R*_SEI_) and charge-transfer reactions (*R*_ct_), respectively [8, 63–66]. The straight line at the low-frequency region was associated with the solid-state diffusion of Li (D_Li_) within siloxene [63, 65–67]. After delithiation (Fig. 6b), the two semicircles became smaller and overlapped more than those in the lithiated state, indicating that Li-ion migration within the SEI layers and the charge transfer reactions exhibited comparable relaxation time constants in the delithiation state [66], presumably originating from the enhanced kinetics of both processes. Notably, the Nyquist plots of SX/A (Fig. S9) demonstrated a similar pattern with those of n-SX/A.

By fitting the measured data to the equivalent circuit (inset of Fig. 6a), we extracted the *R*_SEI_ (Fig. 6c) and *R*_ct_ (Fig. 6d) of n-SX/A and SX/A in their lithiated and delithiated states over a series of cycles. In the case of R_SEI_ in the lithiated state, SX/A showed a monotonic decrease with cycling, whereas n-SX/A displayed a steep increase up to the 10th cycle, followed by a gradual decrease. This observed anomaly in n-SX/A—a steep increase followed by a gradual decrease—can be presumably attributed to the aggressive electrochemical reduction of the electrolyte, driven by the high electron activity of n-SX/A during initial cycles, leading to the formation of unstable, poorly crystalline SEI layers. After this initial consumption period, the amount of available electrolyte molecules for reduction decreases, resulting in the stabilization of the SEI layers. Starting from the 50th cycle, the *R*_SEI_ values and their trends for both electrodes converged. In contrast, in the delithiated state, the two electrodes demonstrated nearly identical *R*_SEI_ values over the entire cycling process, except for some trivial discrepancies at the 25th cycle. Thus, it can be inferred that P doping barely influenced Li-ion migration within the SEI layers. In contrast to the *R*_SEI_, the two electrodes exhibited significantly different *R*_ct_ values in both the lithiated and delithiated states, with those of n-SX/A being consistently smaller than those of SX/A up to the 100th cycle. This observation confirmed that the enhanced electrical conduction in n-SX can promote the charge transfer reaction rate. Using the plots of Z’ (real part of the impedance) vs. ω^−1/2^ (angular frequency^−1/2^) across the low-frequency region (Figs. 6e and S10), the D_Li_ values of both electrodes were calculated (Fig. 6f). In both the lithiated and delithiated states, n-SX/A demonstrated a solid-state diffusion behavior that was 2–3 times faster than that of SX/A during cycling. The enhanced solid-state diffusion in n-SX/A could be because Li diffusion in the solid is readily promoted by the coupled movement of Li ions and electrons [68], as a higher electrical conductivity can facilitate Li diffusion.

Notably, for both electrodes, the *R*_SEI_ values after lithiation were larger than those after delithiation, likely because of the higher activation energy of Li-ion migration within the SEI layers after lithiation [40]. Similar trends were observed for the *R*_ct_. The charge-transfer reactions of both electrodes after lithiation were more sluggish than those after delithiation. Lithiated siloxenes generally contain defects such as LiSi polymorphs or alloys [9], which increase the interface energy, resulting in poor charge transfer performance [40]. In contrast, solid-state diffusion processes within lithiated n-SX/A and SX/A occurred by 2–3 orders of magnitude faster than those within the delithiated electrodes. The most prominent explanation for this result was that in the lithiated state, the presence of local defects, such as LiSi polymorphs and alloys, may enhance Li diffusion by reducing the free energy for migration [9, 69].

### Surface Chemistry after Cycling

**Fig. 7:**
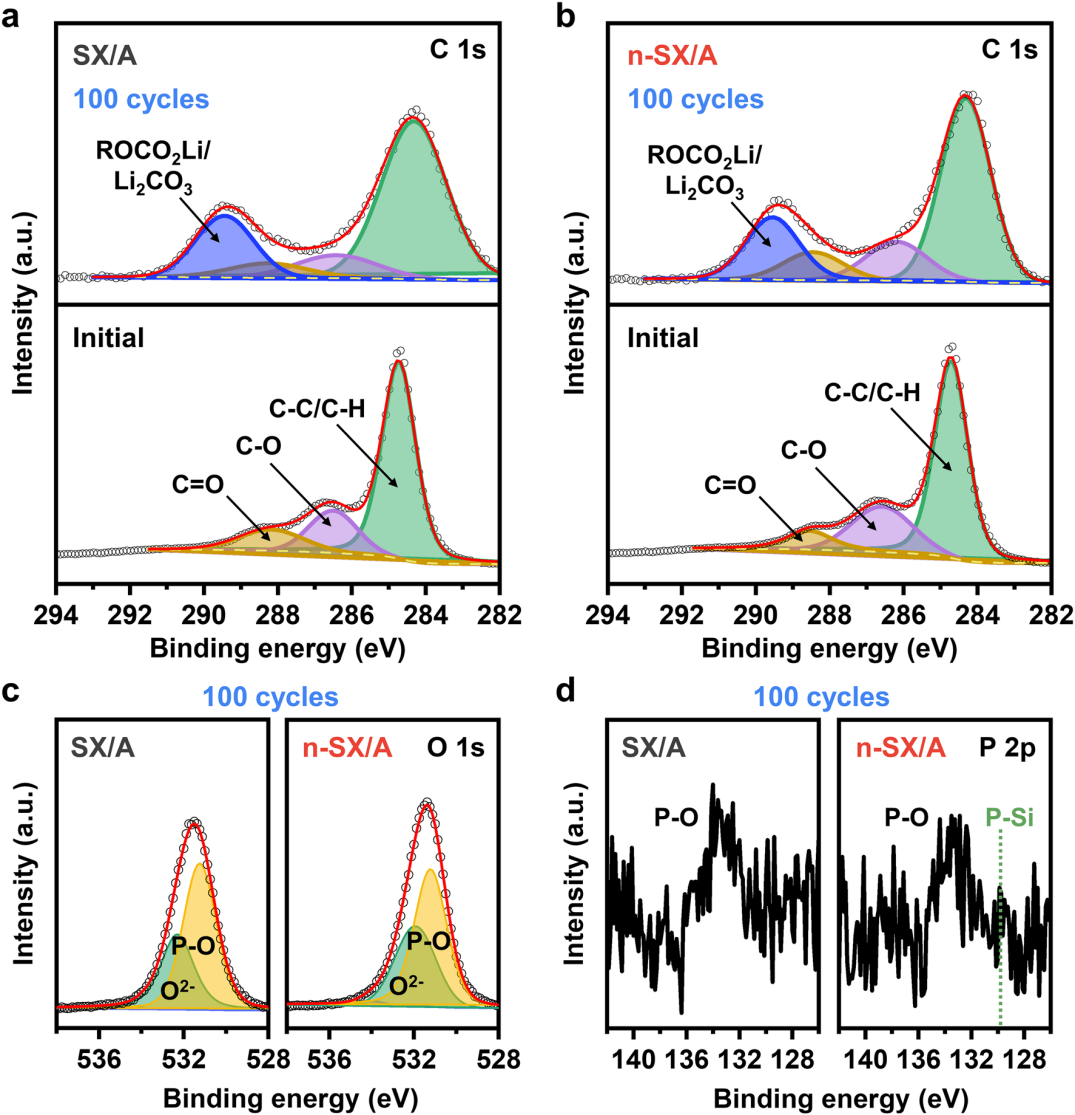
Characterization of SEI layers formed after cycling. XPS high-resolution C 1*s* spectra of **a** SX/A and **b** n-SX/A at their initial states (bottom panels) and after 100 cycles at 1000 mA g^−1^ (top panels). XPS high-resolution **c** O 1*s* and **d** P 2*p* spectra of SX/A and n-SX/A after 100 cycles

Understanding electrode surfaces after cycling enables the design of better materials. Figure 7a, b exhibits the high-resolution XPS C 1*s* spectra of SX/A and n-SX/A, respectively, in their initial states and after 100 cycles. Before cycling, both electrodes exhibited three peaks corresponding to C=O (288.2 eV), C–O (286.5 eV), and C–C/C–H bonds (284.8 eV) [70–72]. These bonds were attributed to the presence of alginate binders and super P-conducting agents [71, 72]. After 100 cycles, an additional peak emerged at 289.5 eV for both electrodes, originating from the CO_3_ bonds of the ROCO_2_Li/Li_2_CO_3_ components formed in the SEI layers [71–75]. The ratio of the integrated intensities of these peaks between SX/A and n-SX/A was ~ 1 (8616/8560), implying that the amounts of ROCO_2_Li/Li_2_CO_3_ in the SEI layers of both electrodes were similar after 100 cycles. Figure 7c shows the high-resolution XPS O 1*s* spectra of both electrodes, which were cycled 100 times. In contrast to what was observed before cycling (Fig. 3c), the SX/A spectrum after 100 cycles exhibited the emergence of a new peak at 531.4 eV, representing the existence of a P–O bond. Additionally, for n-SX/A, the intensity of this peak was greatly enhanced after 100 cycles compared to that before cycling (Fig. 3c). These observations suggest the formation of a Li_x_POF_y_ component in the SEI layer [76]. The comparable integrated intensities of these peaks in SX/A and n-SX/A indicated similar Li_x_POF_y_ contents in the SEI layers of the two electrodes, with the P 2*p* XPS profiles in Fig. 7d further supporting this conclusion. The peak associated with the P–O bond at 133.5 eV was also observed in SX/A’s spectrum after cycling as well.

The P–O peaks in the XPS O 1*s* and P 2*p* spectra of a pristine n-SX (Fig. 3c, d) are attributed to the presence of O_3_≡P units. In contrast, as the surfaces of an n-SX/A electrode after 100 cycles are completely covered with SEI layers, the P–O peaks in its XPS spectra for O 1*s* and P 2*p* (Fig. 7c, d) originate from the presence of Li_x_POF_y_ components in the SEI layers. The intensity of the P–O peak in the O 1*s* spectrum for the pristine n-SX (~ 8000) (Fig. 3c) is ~ 3.3 times smaller than that for the 100-time-cycled n-SX/A electrode (~ 26,000) (Fig. 7c), indicating that the number density of O_3_≡P units in n-SX is lower than that of Li_x_POF_y_ in the cycled n-SX/A electrode. This is reasonable because the P content in n-SX is low (~ 0.05 at%). Typically, there is a large discrepancy between the peak intensities of O 1*s* and P 2*p* spectra [77–79], owing to the XPS photoionization cross-section (i.e., Scofield factor) of P 2*p* (0.4) being ~ 7.5 times smaller than that of O 1*s* (2.92) [80]. As expected, the P–O peak intensity in the P 2*p* spectrum of the cycled n-SX electrode remains only at ~ 290 (Fig. 7d), which is much weaker than that in its O 1*s* spectrum (Fig. 7c). This behavior is also observed for the pristine n-SX; the peak intensity of the P 2*p* spectrum is only ~ 650 (Fig. 3d). However, the decrease in the peak intensity from O 1*s* to P 2*p* for the pristine n-SX (8000/650≈12) is not as large as that for the cycled n-SX/A (26,000/290≈90). The reason for this may be because the O_3_≡P units in n-SX possess the clearly defined chemical bonds and composition—characteristics that could improve the sensitivity to XPS measurements [81], whereas Li_x_POF_y_ components in the SEI layers have the ambiguous chemical bonds and composition. Consequently, after cycling, the P–O peak intensity decreases in the P 2*p* spectra, but it increases in the O 1*s* spectra.

The morphologies of SX/A and n-SX/A after 100 cycles are shown in Fig. S11. Regardless of whether P was incorporated or not, the layered structures remained intact without any structural degradations, suggesting that Li-ion storage primarily relies on the intercalation mechanism [6]. Compared to the peaks before cycling, in the XPS Si 2*p* spectra of both SX/A and n-SX/A after cycling (Fig. S12), the peaks at the higher binding energy (103.3 eV) were enhanced, while those at the lower binding energy (99.5 eV) were suppressed. After cycling, the Z_Si_^eff^ values of SX and n-SX increased by 10% (2.22) and 14% (2.82), respectively. This increase was presumably due to the continuous exposure to the O-containing electrolyte during lithiation/delithiation [6, 9].

## Conclusions

In this study, enhanced Li-ion storage was demonstrated using an n-SX anode that can be fabricated through a nondestructive low-temperature P-doping process, without compromising its 2D layered morphology or Kautsky-type crystal structure. The selective substitution of P dopants for Si atoms in SX was confirmed, and ascribed to the difference in the positive polarization level among Si atoms in different tetrahedral building blocks. The oxidation state difference between P^5+^ and Si^4+^ in the crystal, combined with the creation of H vacancies after doping, enabled the generation of two delocalized electrons per P dopant. Consequently, improved storage capacity and rate performance were achieved for the n-SX anode in LIBs, which conjointly originated from the enhanced kinetics of the charge-transfer reaction, solid-state diffusion, and electronic conduction processes.

The fabrication and systematic investigation of n-SX offer an unprecedented route for engineering advanced SX components for LIB anodes. The doping mechanism revealed in this study can be utilized when designing the incorporation of dopants into other functional 2D materials. Additional computational research could explore the fundamentals of the atomistic interactions between Li ions and dopants in SX. We expect that n-SX with controlled dopant concentrations can offer wide applicability in broader research areas, such as Li–S batteries, supercapacitors, photoelectrochemistry, and optoelectronics, where facile electron transport is required.

## Supplementary Information

Below is the link to the electronic supplementary material.

Supplementary file1 (DOCX 2198 KB)
