# Supplementary material for: Boosted Lithium-Ion Transport Kinetics in n-Type Siloxene Anodes Enabled by Selective Nucleophilic Substitution of Phosphorus
Source: Nanomicro Lett. 2024 Jun 17;16:219. doi: 10.1007/s40820-024-01428-y (PMC11183009; doi:10.1007/s40820-024-01428-y)
Supplement: Supplementary file 1 — Supplementary file1 (DOCX 2198 KB) [file 40820_2024_1428_MOESM1_ESM.docx]

Supporting Information for

**Boosted Lithium-Ion Transport Kinetics in n-Type Siloxene Anodes Enabled by Selective Nucleophilic Substitution of Phosphorus**

Se In Kim^1^, Woong-Ju Kim^1^, Jin Gu Kang^2,^ *, and Dong-Wan Kim^1,^ *

^1^School of Civil, Environmental and Architectural Engineering, Korea University, Seoul 02841, South Korea

^2^Nanophotonics Research Center, Korea Institute of Science and Technology, Seoul, 02792, South Korea

*Corresponding authors. E-mail: [dwkim1@korea.ac.kr](mailto:dwkim1@korea.ac.kr) (Dong-Wan Kim); [lucid1@kist.re.kr](mailto:lucid1@kist.re.kr) (Jin Gu Kang)

**Supplementary Note S1**

Using the elemental analysis results obtained via ICP-OES, we determined the doping concentrations of the doped SX samples. The number density of the SX molecules (N_SX_) is:

$N_{SX}=\frac{N_{A}\rho_{SX}}{M_{W}}$ **(S1)**

where N_A_ is Avogadro’s number (6.02×10^23^ mol^-1^), ρ_SX_ is the density of SX (1.54 g cm^-3^) [S1–S3], and M_W_ is the molecular weight of SX (222 g mol^-1^). Thus, the doping concentration (N_D_, in atoms cm^-3^) can be calculated by:

  $N_{D}=\frac{6N_{A}\rho_{SX}}{M_{W}}\frac{x_{P}}{x_{Si}+x_{P}}$ **(S2)**

where a factor of 6 is the number of Si sites in one formula unit of SX (i.e., Si_6_O_3_H_6_), and x_Si_ and x_P_ are the measured atomic percentages of Si and P, respectively.

**Supplementary Figures and Tables**

**
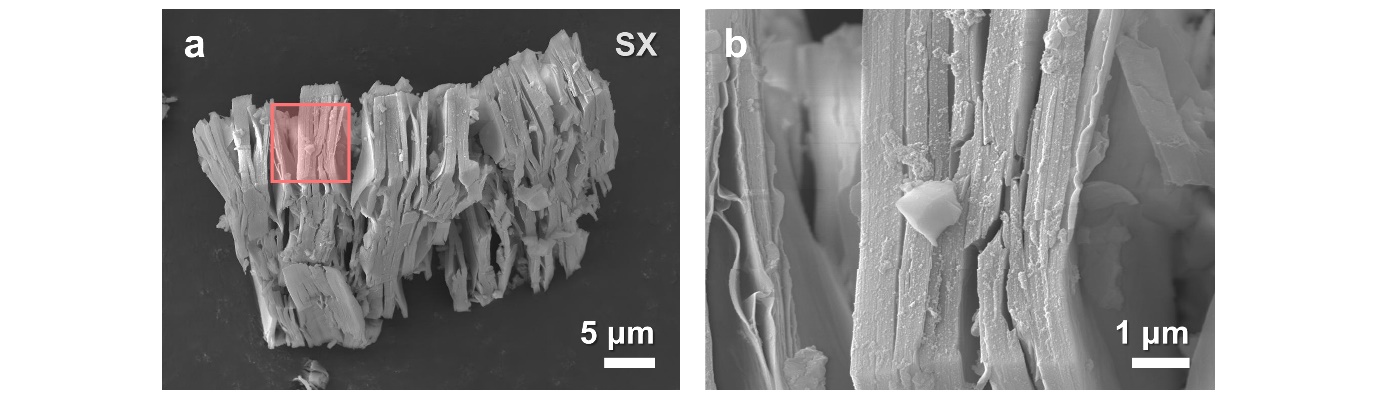
**

**Fig. S1 a** Low- and **b** high-magnification SEM images of SX. **b** Magnified image of the square region in **a**

**
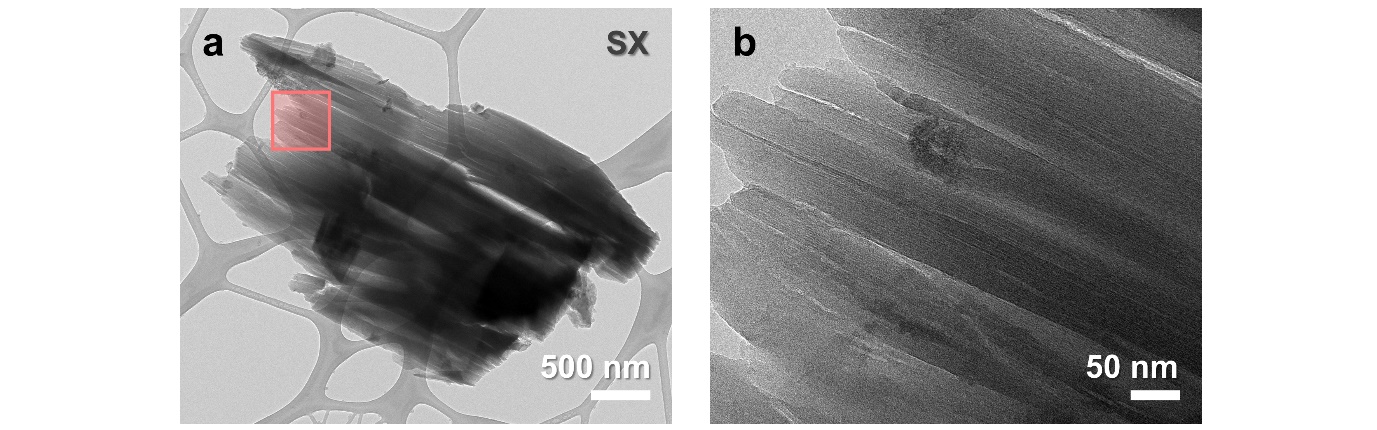
**

**Fig. S2 a** Low- and **b** high-magnification TEM images of SX. **b** Magnified image of the square region in **a**

**
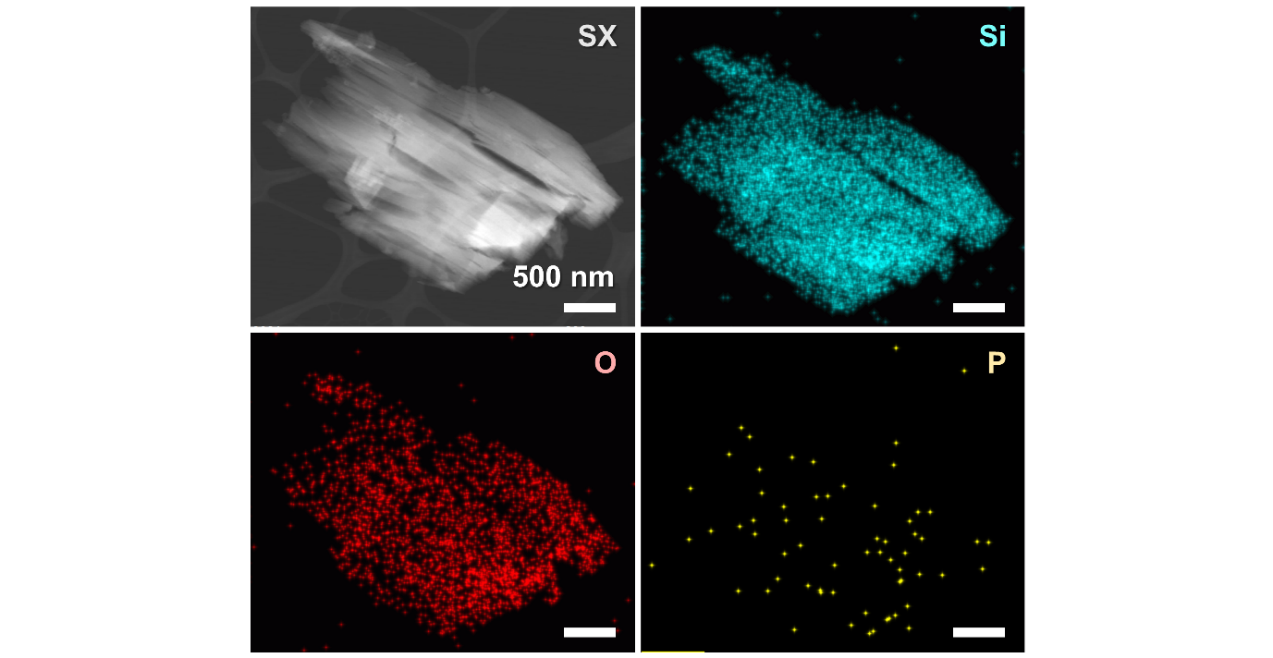
**

**Fig. S3** EDX elemental mapping of Si, O, and P in SX

**
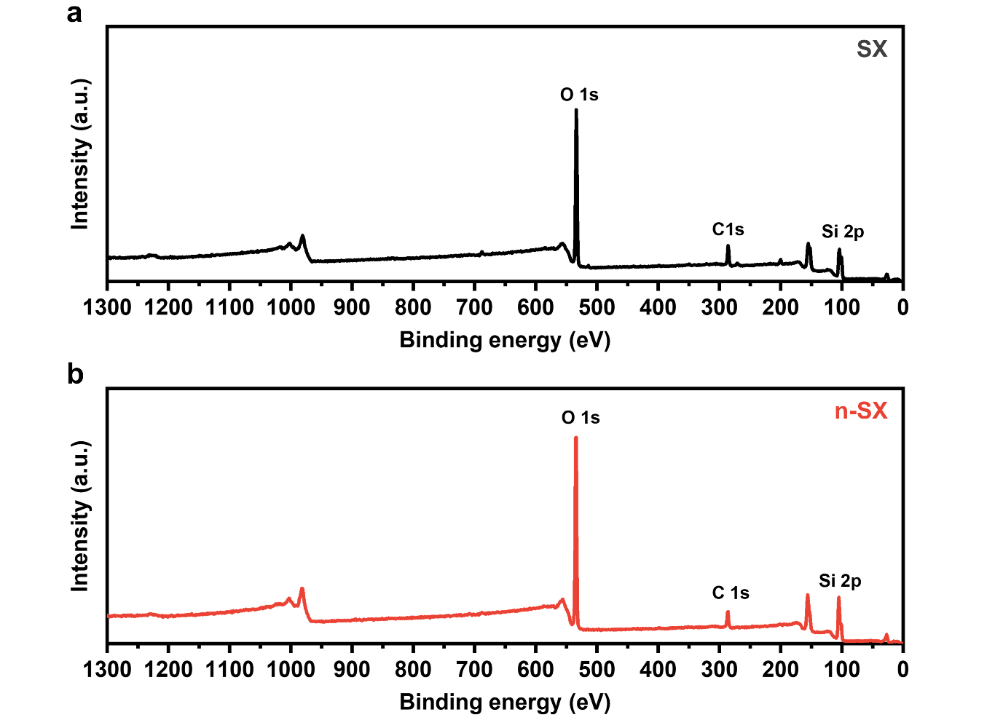
**

**Fig. S4** Survey XPS spectra of **a** SX and **b** n-SX

**
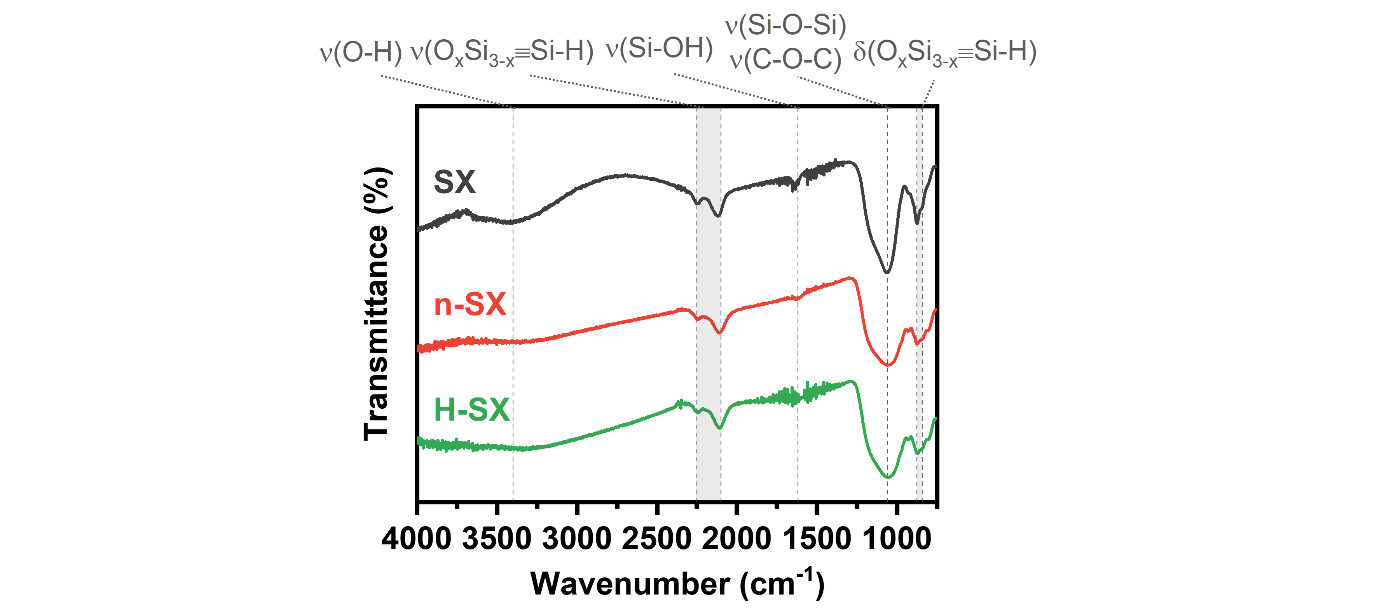
**

**Fig. S5** Full-range FTIR transmittance spectra of SX, n-SX, and H-SX. Peaks are indexed with their corresponding vibrational modes on the image top

**
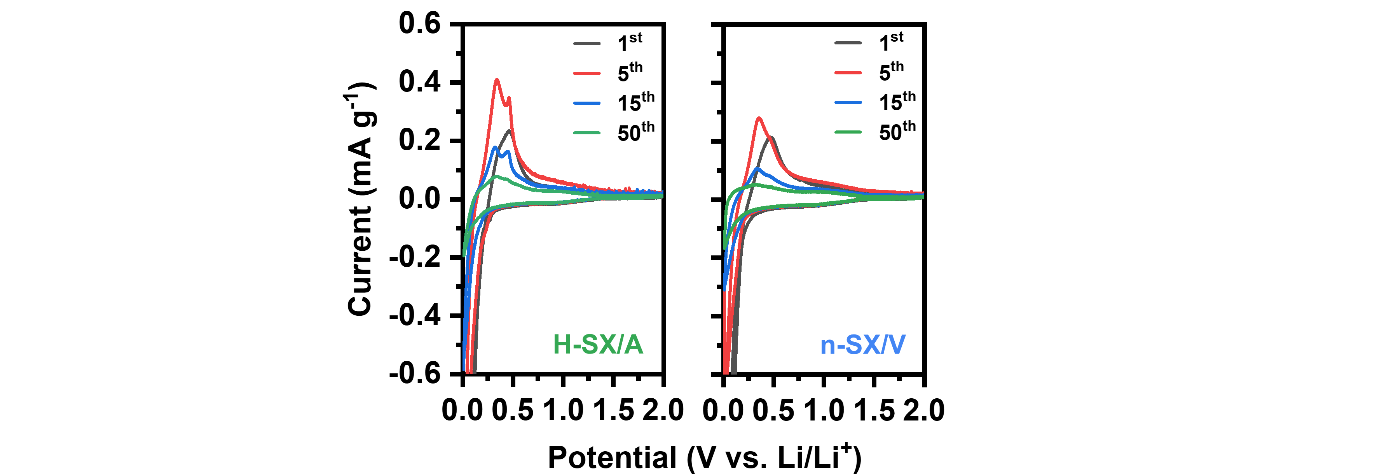
**

**Fig. S6** CV curves of H-SX/A (left panel) and n-SX/V (right) at a scan rate of 0.1 mV s^-1^ during first, 5^th^, 15^th^, and 50^th^ cycles

**
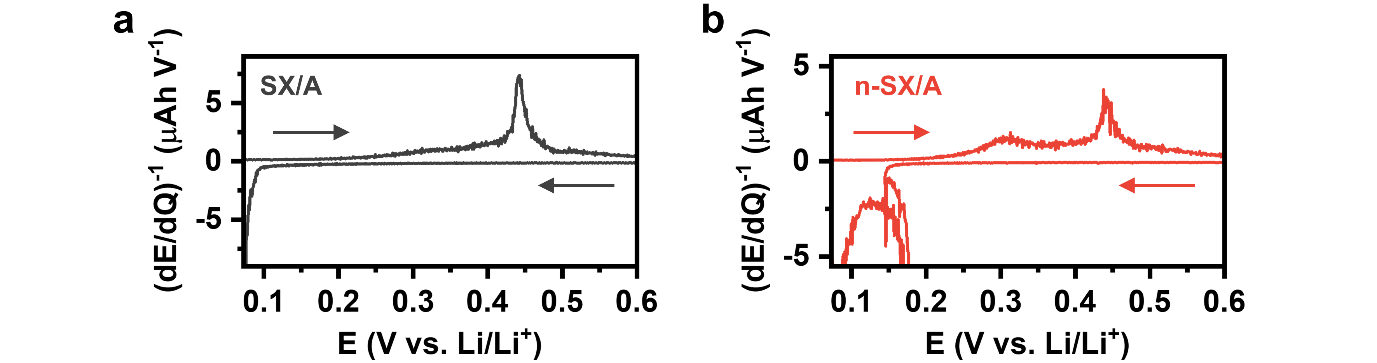
**

**Fig. S7** Plots of (dE/dQ)^-1^ vs. E for **a** SX/A and **b** n-SX/A in the 0.01- 0.6 V range, acquired from their galvanostatic voltage curves at 200 mA g^-1^

**
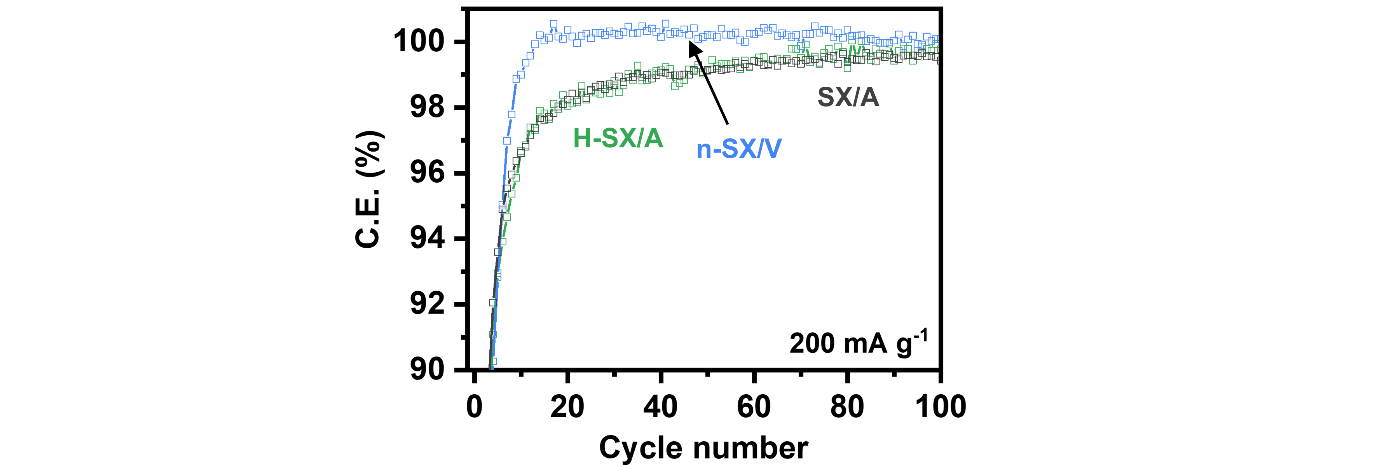
**

**Fig. S8** C.E. of SX/A, H-SX/A, and n-SX/V at 200 mA g^-1^

**
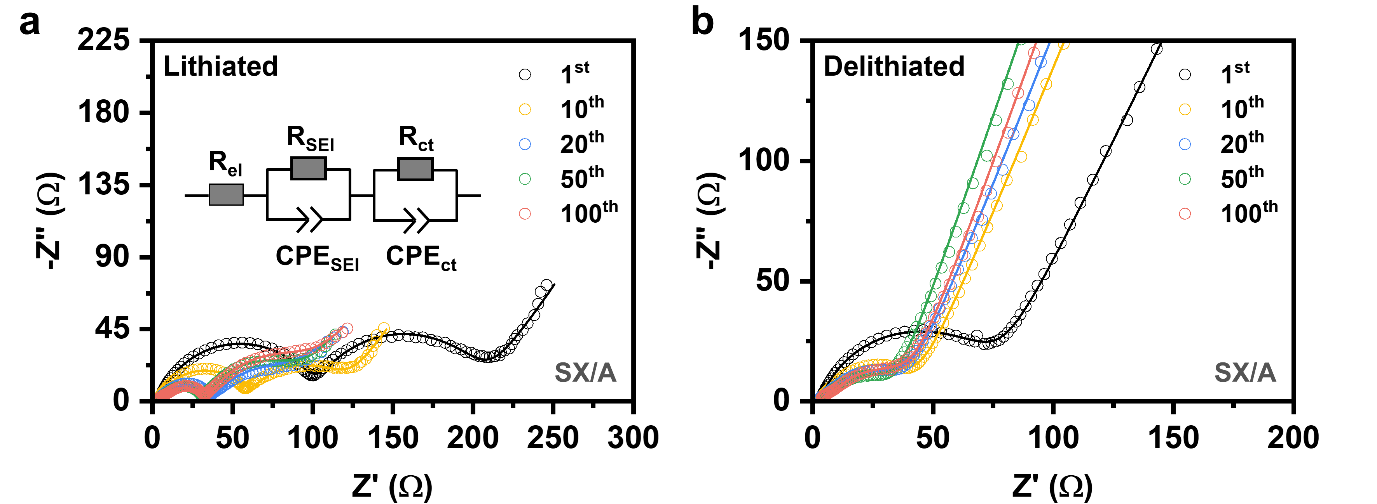
**

**Fig. S9** Nyquist plots of SX/A at its **a** lithiated and **b** delithiated state during first, 10^th^, 20^th^, 50^th^, and 100^th^ cycles. Open circles and solid lines denote the measured data and fitted results, respectively

**
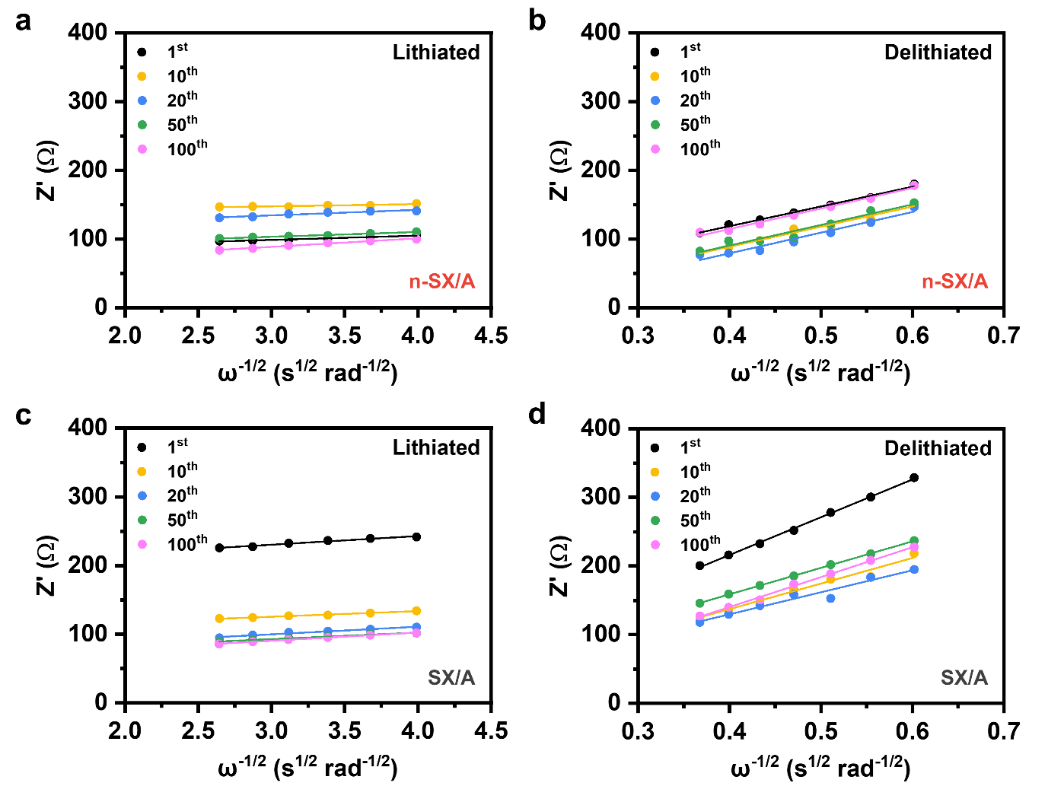
**

**Fig. S10.** Plots of Z’ vs. ω^−1/2^ for **a,b** n-SX/A and **c,d** SX/A at their lithiated **a,c** and delithiated **b,d** states over a series of cycles (first, 10^th^, 20^th^, 50^th^, and 100^th^). Measured data were fitted with linear-regression to extract the Li diffusion coefficients

**
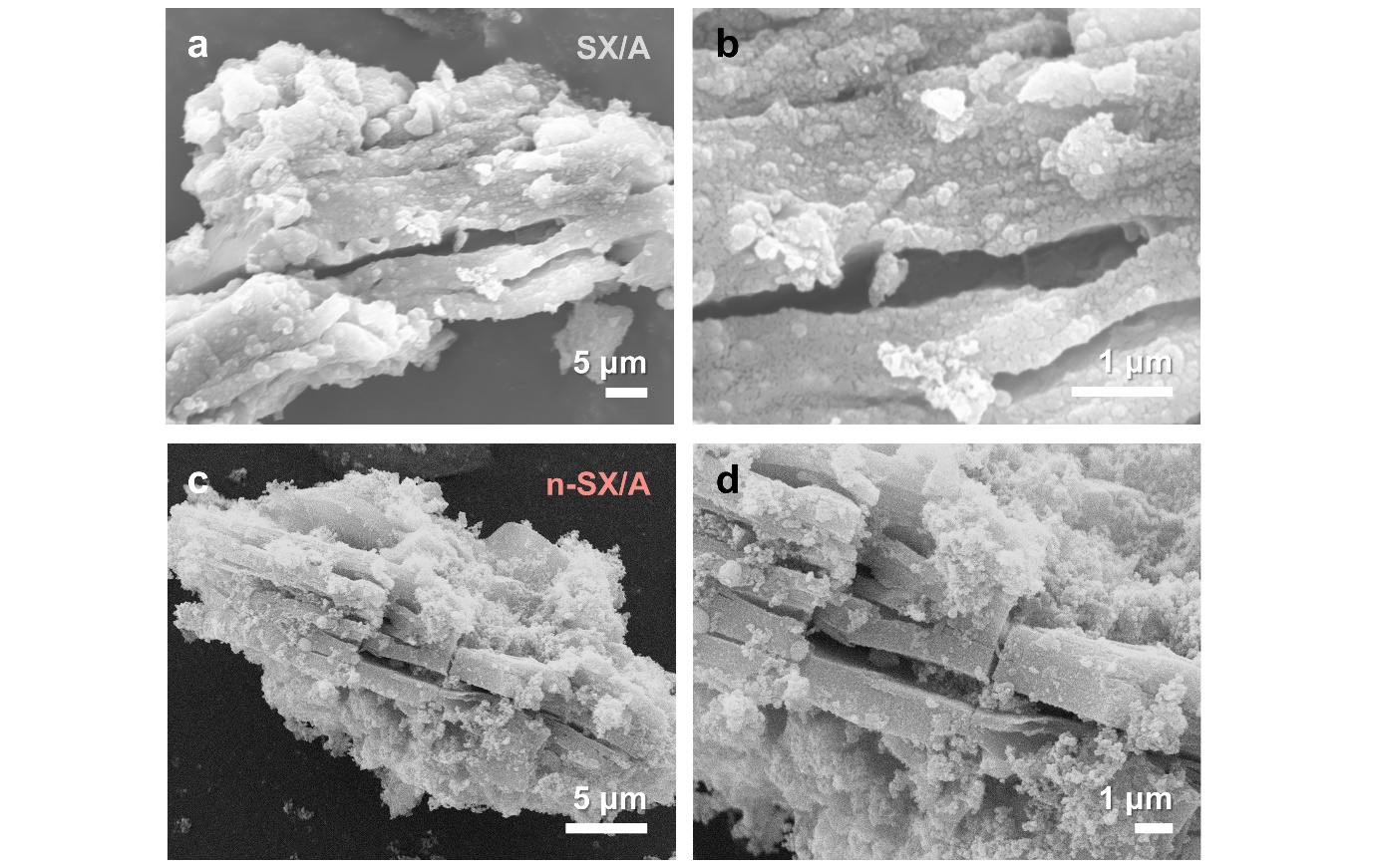
**

**Fig. S11** Post-cycling **a** low- and **b** high-magnification SEM images of SX/A and **c** low- and **d** high-magnification SEM images of n-SX/A (after 100 cycles)

**
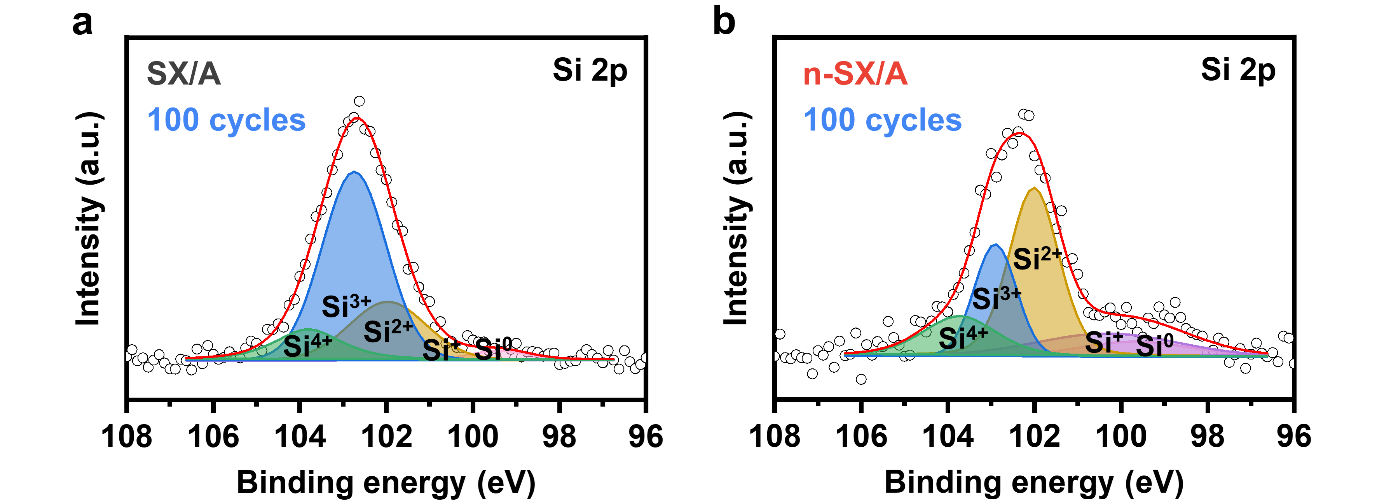
**

**Fig. S12** High-resolution XPS Si 2p spectra of **a** SX/A and **b** n-SX/A after 100 cycles

**Table S1** ICP-OES elemental analysis results and doping concentrations

| **Electrode** | **Doping process condition** | | **Content (at%)** | | **Doping**  **concentration (atoms cm^-3^)** |
| --- | --- | --- | --- | --- | --- |
|  | **SX:NaH_2_PO_2_** | **Ar flow rate (sccm)** | **Si** | **P** |  |
| SX | N.A. | N.A. | 49.6±0.001 | 0 | 0 |
| n-SX | 1:2 | 100 | 51.7±0.003 | 0.05±0.02 | 2.4×10^19^ |
| n-SX-101 | 1:10 | 100 | 56.0±0.02 | 0.08±0.04 | 3.6×10^19^ |
| n-SX-102 | 1:10 | 200 | 55.4±0.005 | 0.15±0.004 | 6.7×10^19^ |
| n-SX-103 | 1:10 | 300 | 49.4±0.001 | 0.16±0.02 | 8.1×10^19^ |

**Table S2** Key electrochemical properties of the electrodes at 2000 mA g^-1^

| **Electrodes** | **Activation**  **cycle** | **Post-activation**  **capacity (mAh g^-1^)** | **Capacity**  **retention (%)** |
| --- | --- | --- | --- |
| n-SX | 60 | 552 | 44 |
| n-SX-101 | 25 | 621 | 39 |
| n-SX-102 | 25 | 594 | 73 |
| n-SX-103 | 15 | 756 | 56 |
| 1n3S | 60 | 329 | 67 |
| 1n1S | 30 | 315 | 56 |
| 3n1S | 14 | 413 | 45 |
| SX | 230 | 153 | 90 |

**Table S3** Performance summary of siloxene-based electrodes for lithium ion batteries

| **Material** | **Engineering** | | **CD**  **(mA g^-1^)** | **Capacity**  **(mAh g^-1^)** | **Cycle**  **number** | **Retention**  **(%)** | **Refs.** |
| --- | --- | --- | --- | --- | --- | --- | --- |
|  | **Intrinsic** | **External** |  |  |  |  |  |
| **n-SX-102** | **Doping** | N.A. | **2000** | **594** | **500** | **73** | **This work** |
| SX/SiO_x_ | Oxidization | N.A. | 750 | ~700 | 300 | 98.6 | [S2] |
| SX/SiO_x_ | Oxidization | N.A. | 1000 | 506 | 800 | ~99 | [S4] |
| SX | Oxidization | N.A. | 1000 | 750 | 200 | 77 | [S3] |
| SX | N.A. | Binder  selection | 1000 | 231 | 1400 | 55 | [S5] |
| rGO-SX | N.A. | Aerogel-mediated | 1000 | 243 | 1000 | 99.5 | [S6] |
| COF-SX | N.A. | Surface  modification | 2000 | 584 | 1000 | ~99 | [S7] |
| G-SX | N.A. | Surface modification | 4100 | 1040 | 1000 | 70 | [S8] |
| LM-SX | N.A. | Matrix-embedded | 2000 | 847 | 300 | ~99 | [S9] |
| Li-SX | N.A. | Prelithiated | 5000 | ~680 | 724 | 80 | [S10] |
| SX | N.A. | N.A. | 193 | 1845 | 58 | 79 | [S11] |

rGO: reduced graphene oxide; COF: covalent organic framework; G: graphene; LM: liquid metal

**Supplementary References**

1. S. Yamanaka, H. Matsu-ura, M. Ishikawa, New deintercalation reaction of calcium from calcium disilicide. Synthesis of layered polysilane. Mater. Res. Bull. **31**, 307-316 (1996). [https://doi.org/10.1016/0025-5408(95)00195-6](https://doi.org/https://doi.org/10.1016/0025-5408(95)00195-6)
2. R. Fu, Y. Li, Y. Wu, C. Shen, C. Fan et al., Controlling siloxene oxidization to tailor SiO_x_ anodes for high performance lithium ion batteries. J. Power Sources. **432**, 65-72 (2019). [https://doi.org/10.1016/j.jpowsour.2019.05.071](https://doi.org/https://doi.org/10.1016/j.jpowsour.2019.05.071)
3. D. J. Arnot, W. Li, D. C. Bock, C. A. Stackhouse, X. Tong et al., Low-oxidized siloxene nanosheets with high capacity, capacity retention, and rate capability in lithium-based batteries. Adv. Mater. Interfaces **9**, 2102238 (2022). [https://doi.org/10.1002/admi.202102238](https://doi.org/https://doi.org/10.1002/admi.202102238)
4. Y. Ren, X. Yin, L. Xiang, R. Xiao, H. Huo et al., Layer stacked SiO_x_ microparticle with disconnected interstices enables stable interphase and particle integrity for lithium-ion batteries. J. Energy Chem. **86**, 300-307 (2023). [https://doi.org/10.1016/j.jechem.2023.07.023](https://doi.org/https://doi.org/10.1016/j.jechem.2023.07.023)
5. S. I. Kim, W.-J. Kim, J. G. Kang, D.-W. Kim, Intermolecular interaction engineering to enhance lithium-ion storage in two-dimensional oxidized silicon nanosheet anodes. Chem. Eng. J. **467**, 143364 (2023). [https://doi.org/10.1016/j.cej.2023.143364](https://doi.org/https://doi.org/10.1016/j.cej.2023.143364)
6. S. A. Alomari, D. P. Dubal, J. MacLeod, N. Motta, Three-dimensional nitrogen-doped rGO-siloxene nanocomposite anode for Li-ion storage. Appl. Surf. Sci. **624**, 157099 (2023). <https://doi.org/10.1016/j.apsusc.2023.157099>
7. Y. Zhang, Y. Wu, Y. An, C. Wei, L. Tan et al., Ultrastable and high-rate 2D siloxene anode enabled by covalent organic framework engineering for advanced lithium-ion batteries. Small Methods. **6**, 2200306 (2022). [https://doi.org/10.1002/smtd.202200306](https://doi.org/https://doi.org/10.1002/smtd.202200306)
8. K. T. Kumar, M. J. Kumar Reddy, G. S. Sundari, S. Raghu, R. A. Kalaivani et al., Synthesis of graphene-siloxene nanosheet based layered composite materials by tuning its interface chemistry: an efficient anode with overwhelming electrochemical performances for lithium-ion batteries. J. Power Sources. **450**, 227618 (2020). [https://doi.org/10.1016/j.jpowsour.2019.227618](https://doi.org/https://doi.org/10.1016/j.jpowsour.2019.227618)
9. Y. Zhang, L. Tan, Y. Wu, Y. An, Y. Liu et al., Self-healing and ultrastable anode based on room temperature liquid metal reinforced two-dimensional siloxene for high-performance lithium-ion batteries. Appl. Mater. Today **26**, 101300 (2022). [https://doi.org/10.1016/j.apmt.2021.101300](https://doi.org/https://doi.org/10.1016/j.apmt.2021.101300)
10. H. Shen, Y. An, Q. Man, J. Wang, C. Liu et al., Controlled prelithiation of siloxene nanosheet anodes enables high performance 5 V-class lithium-ion batteries. Chem. Eng. J. **454**, 140136 (2023). [https://doi.org/10.1016/j.cej.2022.140136](https://doi.org/https://doi.org/10.1016/j.cej.2022.140136)
11. L. C. Loaiza, L. Monconduit, V. Seznec, Siloxene: a potential layered silicon intercalation anode for Na, Li and K ion batteries. J. Power Sources **417**, 99-107 (2019). [https://doi.org/10.1016/j.jpowsour.2019.02.030](https://doi.org/https://doi.org/10.1016/j.jpowsour.2019.02.030)
